# Dysregulation and prometastatic function of glycosyltransferase C1GALT1 modulated by cHP1BP3/ miR-1-3p axis in bladder cancer

**DOI:** 10.1186/s13046-022-02438-7

**Published:** 2022-07-21

**Authors:** Zengqi Tan, Yazhuo Jiang, Liang Liang, Jinpeng Wu, Lin Cao, Xiaoman Zhou, Zhihui Song, Zhenyu Ye, Ziyan Zhao, Hui Feng, Zewen Dong, Shuai Lin, Zhangjian Zhou, Yili Wang, Xiang Li, Feng Guan

**Affiliations:** 1 Institute of Hematology, Provincial Key Laboratory of Biotechnology, School of Medicine, Northwest University, Xi’an, 710069 People’s Republic of China; 2 Department of Urology, The Third Affiliated Hospital of Xi’an Jiaotong University, Xi’an, 710068 People’s Republic of China; 3 Department of Urology, The First Affiliated Hospital of Xi’an Jiaotong University, Xi’an, 710061 People’s Republic of China; 4 Key Laboratory of Resource Biology and Biotechnology in Western China, Ministry of Education, Provincial Key Laboratory of Biotechnology, College of Life Sciences, Northwest University, 229 Taibai North Road, Xi’an, 710069 Shaanxi China; 5 Department of Oncology, The Second Affiliated Hospital of Xi’an Jiaotong University, Xi’an, 710004 People’s Republic of China; 6 Institute for Cancer Research, School of Basic Medical Science, Health Science Center of Xi’an Jiaotong University, Xi’an, 710061 People’s Republic of China

**Keywords:** C1GALT1, miR-1-3p, circRNA, cHP1BP3, Bladder cancer

## Abstract

**Background:**

Abnormal glycosylation in a variety of cancer types is involved in tumor progression and chemoresistance. Glycosyltransferase C1GALT1, the key enzyme in conversion of Tn antigen to T antigen, is involved in both physiological and pathological conditions. However, the mechanisms of C1GALT1 in enhancing oncogenic phenotypes and its regulatory effects via non-coding RNA are unclear.

**Methods:**

Abnormal expression of C1GALT1 and its products T antigen in human bladder cancer (BLCA) were evaluated with BLCA tissue, plasma samples and cell lines. Effects of C1GALT1 on migratory ability and proliferation were assessed in YTS-1 cells by transwell, CCK8 and colony formation assay in vitro and by mouse subcutaneous xenograft and trans-splenic metastasis models in vivo. Dysregulated circular RNAs (circRNAs) and microRNAs (miRNAs) were profiled in 3 pairs of bladder cancer tissues by RNA-seq. Effects of miR-1-3p and cHP1BP3 (circRNA derived from HP1BP3) on modulating C1GALT1 expression were investigated by target prediction program, correlation analysis and luciferase reporter assay. Functional roles of miR-1-3p and cHP1BP3 on migratory ability and proliferation in BLCA were also investigated by in vitro and in vivo experiments. Additionally, glycoproteomic analysis was employed to identify the target glycoproteins of C1GALT1.

**Results:**

In this study, we demonstrated upregulation of C1GALT1 and its product T antigen in BLCA. C1GALT1 silencing suppressed migratory ability and proliferation of BLCA YTS-1 cells in vitro and in vivo. Subsets of circRNAs and miRNAs were dysregulated in BLCA tissues. miR-1-3p, which is reduced in BLCA tissues, inhibited transcription of C1GALT1 by binding directly to its 3′-untranslated region (3′-UTR). miR-1-3p overexpression resulted in decreased migratory ability and proliferation of YTS-1 cells. cHP1BP3 was upregulated in BLCA tissues, and served as an miR-1-3p “sponge”. cHP1BP3 was shown to modulate migratory ability, proliferation, and colony formation of YTS-1 cells, and displayed tumor-suppressing activity in BLCA. Target glycoproteins of C1GALT1, including integrins and MUC16, were identified.

**Conclusions:**

This study reveals the pro-metastatic and proliferative function of upregulated glycosyltransferase C1GLAT1, and provides preliminary data on mechanisms underlying dysregulation of C1GALT1 via miR-1-3p / cHP1BP3 axis in BLCA.

**Supplementary Information:**

The online version contains supplementary material available at 10.1186/s13046-022-02438-7.

## Background

Glycosylation is a complex and commonly occurring type of protein modification that promotes functional plasticity by increasing protein heterogeneity [1, 2]. Normal O-glycosylation, one form of glycosylation, is essential for proper development [2] and cellular differentiation [3]; on the other hand, dysregulation of O-glycans contributes to a variety of pathogenic processes, including inflammation [4], tumorigenesis [5], and metastasis [6]. Addition of N-acetylgalactosamine (GalNAc) to serine or threonine, catalyzed by polypeptide GalNAc transferases (ppGalNAcTs), results in formation of Tn antigen (GalNAcα-O-Ser/Thr). O-glycans based on Tn antigen are extended to various complex, branched structures by sequential glycosyltransferase reactions. Core 1 β1,3-galactosyltransferase (C1GALT1; also termed T-synthase) transfers galactose (Gal) to GalNAc to form Galβ1-3GalNAcα1-Ser/Thr (core 1 O-glycan structure, termed T antigen). Core 1 structure is the precursor for subsequent extension and maturation of mucin-type O-glycans.

Expression of truncated O-glycans, such as sialyl-Tn (STn) and Tn antigens, is frequently observed in gastric, hepatic, and some other types of cancer, and is correlated with cancer aggressiveness and progression [7, 8]. Truncated O-glycans alter antigenic characters and physiological functions of glycoproteins, and promote invasiveness, chemotherapy resistance and metastasis of cancer cells [9, 10]. Some of them have been developed as small-molecule inhibitors [11] and carbohydrate vaccines for application in cancer therapy [12]. Better understanding of how truncated O-glycans are regulated may therefore lead to improved immunotherapeutic strategies [13].

C1GALT1 is localized in Golgi apparatus, and its enzymatic activity depends on an unusual molecular chaperone termed “Cosmc” (core 1 β3GalT specific molecular chaperone). C1GALT1 is commonly upregulated during tumorigenesis, including development of hepatocellular carcinoma [8], breast cancer [14], and some other types of cancer [15]. C1GALT1 has a modulatory effect on expression of target glycoproteins such as MUC16 [16], Muc5Ac, and MUC1 [17], and regulates progression of certain types of cancer. In gastric cancer, it promoted phosphorylation of EPHA2 and enhanced soluble Ephrin A1-mediated migration by modifying EPHA2 O-glycosylation [18]. In pancreatic adenocarcinoma, its knockdown suppressed focal adhesion kinase (FAK) phosphorylation at Y397/Y925, and reduced cell/ extracellular matrix adhesion by modifying integrin αv [15]. A loss of C1GALT1 resulted in a Tn enrichment on CD44, which activated ERK/NF-κB signaling and facilitated the cancer stem cell features in pancreatic cancer cells [19]. However, C1GALT1 functioned differentially in other studies. It is more highly expressed in well-differentiated than in poorly-differentiated pancreatic cancer tissues [16]. Modulation of C1GALT1 expression contributes to alterations of tumorigenesis and metastasis. Mice with knockdown of gastric epithelial C1GALT1 developed spontaneous chronic gastritis and gastric cancer [17]. Downregulation of C1GALT1 suppressed the TrkA expression and promoted malignant behaviors of neuroblastoma cells [20]. To date, few studies have addressed the role of C1GALT1 in bladder cancer (BLCA), and molecular mechanisms that underlie C1GALT1 dysregulation are unclear.

The present study revealed abnormal C1GALT1 levels in BLCA. Normal bladder tissues, in comparison with BLCA tissues, showed differential expression of microRNAs (miRNAs) and circular RNAs (circRNAs). We provide preliminary data on mechanisms underlying dysregulation of C1GALT1 via mRNA/ miRNA/ circRNA axis, and effects of C1GALT1 and corresponding regulatory non-coding RNAs on cell proliferation and migratory ability.

## Methods

### Cell lines and cell culture

Human bladder mucosal epithelial HCV29, human benign non-muscle-invasive BLCA KK47, and highly malignant invasive BLCA YTS-1 cell lines were kindly donated by Dr. S. Hakomori (The Biomembrane Institute, Seattle, WA, USA) [21]. Human uroepithelial SV-HUC-1 and transitional carcinoma T24, J82, 5637, and RT4 cell lines were from the Cell Bank of the Chinese Academy of Sciences (Shanghai). SV-HUC-1 cells were cultured in F12K medium (Invitrogen; Carlsbad, CA, USA) supplemented with 10% fetal bovine serum (FBS; Biological Industries; Beit Haemek, Israel) and 1% antibiotics (100 U/mL penicillin, 100 μg/mL streptomycin sulfates) at 37 °C in 5% CO_2_ atmosphere. Bladder cell lines were cultured in RPMI-1640 medium supplemented with 10% FBS and 1% antibiotics at 37 °C in 5% CO_2_ atmosphere. All cells were used for the experiments between passages 3 and 15.

### Tissue microarray (TMA) analysis

TMA analyses were performed as described previously [22]. TMAs containing BLCA and paracarcinoma tissues were from Shanghai Outdo Biotech Co. In brief, TMAs were deparaffinized, rehydrated, subjected to antigen retrieval, blocked, incubated with biotinylated lectin or primary antibody overnight at 4 °C, rinsed with 1× PBS (0.01 mol/L phosphate buffer containing 0.15 mol/L NaCl, pH 7.4), incubated with HRP-streptavidin or secondary antibody for 1 h at 37 °C, visualized with DAB reagent, stained with hematoxylin, and photographed. Mean optical densities of the TMA staining signals were calculated using Image-Pro Plus software (Media Cybernetics; Silver Spring, MD, USA).

### Total protein extraction

Monolayer cells at 70–80% confluence were rinsed with 1× PBS, added with RIPA buffer (50 mM Tris, pH 7.2, Triton X-100, 0.5% sodium deoxycholate, 0.1% SDS, 150 mM NaCl, 10 mM MgCl_2_, 5% glycerol) containing protease inhibitor (NCM Biotech; Suzhou, China), and incubated for 30 min on ice. BLCA tissues were homogenized in a tissue homogenizer (Jingxin Technology; Shanghai) with 500 μL T-PER Tissue Protein Extraction Reagent (Thermo Scientific; San Jose, CA, USA) containing protease inhibitor. Cell and tissue lysates were sonicated and centrifuged, and supernatants were collected. Protein content was determined by BCA assay (Beyotime Institute of Biotechnology; Haimen, China).

### Western blotting and lectin blotting

Western and lectin blotting were performed as described previously [22]. Proteins were separated by SDS-PAGE and transferred onto PVDF membranes (Bio-Rad; Hercules, CA, USA). Membranes were blocked, probed with primary anti-C1GALT1 antibody (Santa Cruz Biotechnology; Santa Cruz, CA, USA), anti-FGFR3 antibody (Proteintech; Wuhan, China), anti-MUC16 antibody (Proteintech; Wuhan, China), anti-GAPDH antibody (Sigma-Aldrich; St. Louis, MO, USA) or biotin-conjugated T-antigen-reactive lectin (Vector Laboratories; Burlingame, CA, USA) overnight at 4 °C, and incubated with appropriate HRP-conjugated secondary antibody/ streptavidin. Bands were visualized using enhanced chemiluminescence kit with luminescent imaging (Tanon; Shanghai).

### Enzyme-linked immunosorbent assay (ELISA)

ELISA was performed as described previously [22]. In brief, 96-well ELISA plates (Jet Biofil; Guangzhou, Guangdong, China) were coated with serum samples from BLCA patients, blocked with BSA, rinsed with 1× PBS containing 0.05% Tween-20 (PBST), incubated with biotin-labeled peanut agglutinin (PNA), which specifically recognizes T antigen (Vector Labs), added with VECTASTAIN ABC reagent (Vector Labs), and visualized using TMB substrate kit (Beyotime). Optical density at wavelength 450 nm was determined by microplate reader.

### O-glycan analysis by mass spectrometry (MS)

Monolayer cells at 70–80% confluence were rinsed with 1× PBS, scraped, and centrifuged. Pellets were added with lysis buffer (50 mM Tris, pH 7.4, 150 mM NaCl, 1% NP-40, 0.25% sodium deoxycholate, 1% SDS, 1% protease inhibitor), sonicated on ice, centrifuged, and supernatants were collected. Proteins were desalted in a dialysis bag (molecular weight cutoff 8–14 kDa) for 72 h at 4 °C, and lyophilized. Lyophilized proteins were dissolved with 1 mL of 0.1 M NaOH, and incubated with 1 mL of 1 M NaBH_4_ for 15 h at 50 °C. The mixture was acidified to pH 4.0 with 50% acetic acid, desalted with HyperSep Hypercarb SPE Cartridges [23] (Thermo Scientific), and O-glycans were subjected to liquid chromatography/ electrospray ionization/ MS (LC-ESI-MS) (Thermo Scientific). ESI-MS data were acquired as described previously [24]. Samples were injected into ESI source in a 50% methanol stream; parameters: flow rate 100 μL/min, spray voltage 4000 V, sheath gas flow rate 20 arb, auxiliary gas flow rate 5 arb, capillary voltage 37 V, tube lens voltage 250 V, capillary temperature 300 °C.

### Knockdown of C1GALT1 and cHP1BP3

shRNA targeting C1GALT1 or backsplice junction of cHP1BP3 were respectively subcloned into lentivirus vector (Tet-pLKO-puro vector, Addgene; Cambridge, MA, USA) to construct sh-C1GALT1 and sh-cHP1BP3 vector, and shRNA sequences were listed in Table S1. Constructed lentiviral vector and two assistant vectors (pMD2.G and psPAX2) were transfected into HEK293T cells with PEI MAX 40000 (Polysciences; Warrington, PA, USA). Virus particles were collected after 2 days, applied to infect YTS-1 cells, and stable transfectants were selected using puromycin. Transient silencing of C1GALT1 and cHP1BP3 in T24 was performed by transfection of constructed vectors. Silencing of C1GALT1 and cHP1BP3 was induced by doxycycline (Doxy).

### miR-1-3p overexpression

Complementary single-strand DNA oligos encoding pre-miR-1-3p and their flanking 100 sequences were synthesized, annealed, and ligated into Tet-pLKO-puro vector (Addgene; Cambridge, MA, USA) for construction of miR-1-3p overexpression vector. Stable YTS-1 transfectants were established as described in the preceding section. Transient overexpressing of miR-1-3p in T24 was performed by transfection of constructed vectors. Overexpression of miRN1–1-3p was induced by doxycycline (Doxy).

### C1GALT1 overexpression

C1GALT1 overexpression was performed as previously described [25]. Briefly, C1GALT1 was cloned into pLVX-AcGFP1-N1 (Takara; Shiga, Japan) lentiviral vector. Lentivirus was packaged in HEK293T cells and collected. HCV29 cells were established by infecting lentivirus into cells, and stable transfectants were selected by puromycin selection.

### Proliferation assay

Cell proliferation was assayed as described previously [25]. For Edu assay, cells treated with/ without Doxy were incubated with 50 μM Edu for 6 h, fixed with 4% paraformaldehyde, permeabilized with 0.5% Triton X-100, stained with iClick EdU solution (GeneCopoeia; Rockville, MD, USA), rinsed with 1× PBS, and subjected to flow cytometry. For CCK8 assay, cells in 96-well plates were incubated with CCK8 solution (Beyotime) for 4 h, and absorbance at 450 nm was determined using a microplate reader.

### Colony formation assay

Colony formation was assayed as described previously [25]. In brief, cells in 6-well plates (1000 cells/ well) were cultured for 1–2 wk. until colonies were clearly observable. Colonies were rinsed with 1× PBS, fixed with 4% paraformaldehyde, stained with crystal violet solution, and photographed. Acetic acid solution was added to dissolve crystal violet, and absorbance at 595 nm was determined.

### Transwell assay

This assay was performed as described previously [22]. 10^4^ cells suspended in FBS-free medium were inoculated into upper chamber, and medium supplemented with 10% FBS was added into lower chamber. After 24 h culture, cell migrated across the membrane were fixed, stained with crystal violet, and photographed under microscope.

### Patient samples

Sera and tissues from normal subjects and BLCA patients were obtained from the Third Affiliated Hospital of Xi’an Jiaotong University. Written informed consent was obtained from all patients, in accordance with the Declaration of Helsinki. Experiments using human tissues were approved by the Research Ethics Committee of Northwest University.

### RNA sequencing analyses

For circRNA sequencing analysis, total RNAs were isolated from BLCA and adjacent normal tissues using TRIzol reagent kit (Invitrogen) as per manufacturer’s instructions, treated with RNase R, and purified using RNeasy MinElute Cleanup Kit (Qiagen; Valencia, CA, USA). Strand-specific library was constructed using VAHTS Total RNA-seq Library Prep Kit for Illumina, as per manufacturer’s instructions. In brief, circRNAs were enriched by removal of rRNAs, fragmented into short fragments, and reverse-transcribed into cDNA. cDNA was further amplified, and sequenced using Illumina HiSeq 2500 by Gene Denovo Biotechnology (Guangzhou).

For miRNA sequencing analysis, isolated total RNAs (size range 18–30 nt) were enriched by PAGE. 3′ and 5′ adapters were ligated to RNAs, and ligation products were reverse-transcribed by PCR amplification, enriched to generate a cDNA library, and sequenced as above.

### Fluorescence in situ hybridization (FISH)

FISH assay was performed as described previously [26]. FITC-labeled cHP1BP3 and CY5-labeled miR-1-3p probes were designed and synthesized by GenePharma (Shanghai). Hybridization was performed overnight using cHP1BP3 and miR-1-3p probes as per manufacturer’s instructions. Images were acquired by laser confocal microscopy (model TCS SP8 (Leica; Weztlar, Germany). Sequences of cHP1BP3 and miR-1-3p probes for FISH are listed in Table S1.

### RNase R treatment

RNAs from YTS-1 cells were treated with 3 U/μg RNase R (Geneseed; Guangzhou), incubated for 30 min at 37 °C, reverse-transcribed with primers, and detected by reverse transcription quantitative real-time PCR (RT-qPCR), and primers were listed in Table S1.

### Biotin-coupled miRNA capture

Biotin-coupled miRNA pulldown assay was performed as described previously [27, 28]. In brief, 3′-end biotinylated miR-1-3p mimics (GenePharma) were transfected into YTS-1 cells using GP-transfect Mate reagent (GenePharma). Cells were lysed with lysis buffer (20 mM Tris (pH 7.5), 100 mM KCl, 5 mM MgCl_2_, 0.3% NP-40, 50 U of RNase OUT) (Sangon Biotech; Shanghai). Biotinylated RNA complex was pulled down by incubating lysates with streptavidin-coated magnetic beads (Invitrogen) pre-blocked with 1 mg/mL yeast tRNA (Beyotime) and 1 mg/mL BSA. cHP1BP3 levels in bound fractions were evaluated by RT-qPCR.

### Luciferase reporter assay

Sequences of predicted miR-1-3p binding sites of circRNAs and C1GALT1 3′-untranslated regions (3′-UTRs), and corresponding mutants (listed in Table S1), were designed, synthesized, and inserted into luciferase reporter vector psiCHECK-2 (Promega; Madison, WI, USA). Constructed plasmids were co-transfected together with miR-1-3p mimics to YTS-1 cells. Relative luciferase activity was determined using Dual Luciferase Assay Kit (Promega) as per manufacturer’s instructions.

### Identification of proteins with T antigen

Proteins were denatured, reduced, alkylated, digested, and desalted as described previously [22]. Lyophilized peptides (150 μg) were dissolved with 50 mM NH_4_HCO_3_, and incubated with 75 μL PNA-agarose (Vector Labs) overnight at 4 °C. The mixture was rinsed with PBS, and boiled for 10 min to release peptides. Glycopeptides with T antigen were collected by centrifugation and purified using Oasis HLB cartridges (Waters; Milford, MA, USA). Two-dimensional LC-MS/MS and data analysis were performed using LTQ Orbitrap MS (Thermo Fisher), and MaxQuant software program with T antigen as variable modification, as described previously [29, 30].

### Tumor formation in mice

Animal experiments were performed in accordance with guidelines of the Animal Care and Use Committee of Northwest University. Effects of C1GALT1 on BLCA tumor proliferation and metastasis were studied using mouse subcutaneous (s.c.) xenograft, trans-splenic metastasis and popliteal lymphatic nodes (LNs) models.

For mouse s.c. xenograft model, YTS-1 cells with silencing of C1GALT1 or circHP1BP3, or overexpression of miR-1-3p, were grown to 70–80% confluence, suspended in RPMI-1640 medium without FBS (10^7^ cells /mL), and 0.2-mL aliquots were transplanted s.c. into 8-wk-old male BALB/c-nu mice. Tumor size was measured every other day for 3 wk., after which tumors were excised and weighed.

The role of C1GALT1 in BLCA based on blood metastasis to liver was investigated by injecting BLCA cells (10^5^ cells in 100 μL PBS) into spleens of 4- to 6-wk-old mice as described previously [31]. After 4 wk., mice were euthanized, and tumors were excised and weighed.

Tumors, spleens, and livers from each type of experiment were dissected, fixed, and paraffin-embedded for histopathological analysis.

PLNs metastasis model was performed by the injection of 100 μL PBS suspensions of BLCA cells (10^5^ cells in 100 μL PBS) into footpads of 4- to 6-wk-old mice as described previously [32]. After 4 wk., popliteal lymphatic nodes were dissected, fixed, and paraffin-embedded for histopathological analysis.

### Patient-derived xenografts (PDXs) models

PDX tumor was derived from a male patient with papillary urothelial carcinoma. Briefly, three serial passage xenograft tumor pieces of ~ 60 mm were subcutaneously grafted into the 4- to 6-wk-old mice. When tumors grown to ~ 200 mm, the mice were randomly divided into vehicle or itraconazole (ITZ) subgroups. Solvent control or ITZ (20 mg/mL, twice daily) was given orally. Tumor size was measured, after which tumors were excised, weighed and subjected to histopathological analysis.

### Statistical analysis

Data are presented as mean ± SD from three independent experiments unless specified otherwise. Two-tailed Student’s t-test was used for comparison of data sets between two groups, and differences with *p* < 0.05 were considered statistically significant. Notations in figures: *p < 0.05; **p < 0.01; ***p < 0.001.

## Results

### Upregulation of C1GALT1 expression in BLCA

**Fig. 1:**
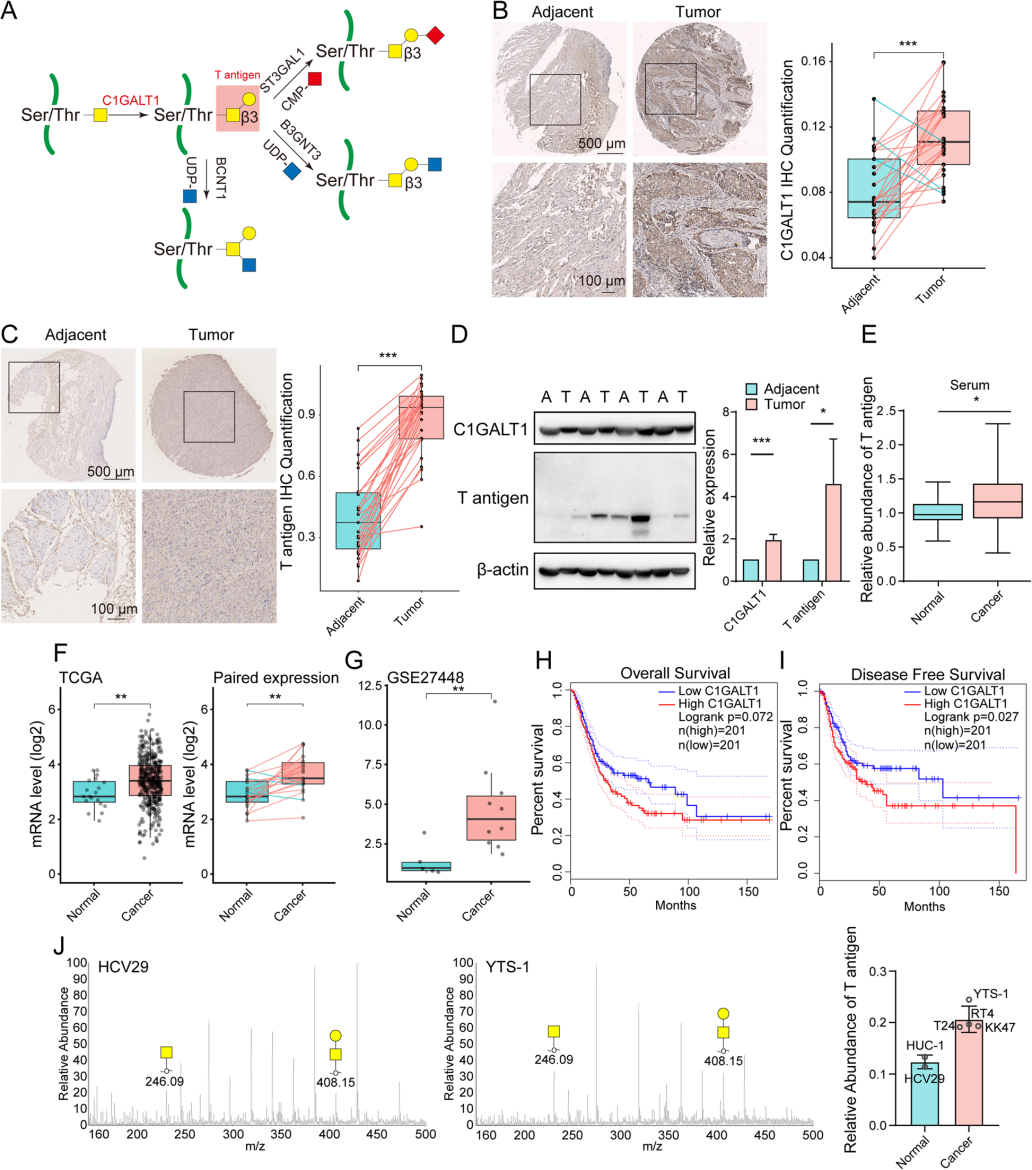
Expression of C1GALT1 and T antigen in BLCA. **A** T antigen production by C1GALT1. **B, C** Differential C1GALT1 expression (**B**) and T antigen levels (**C**) in BLCA, by TMA analysis. Representative immunohistochemical results for paired clinical tissues are shown. ***, *p* < 0.001. **D** Representative western and lectin blotting results for C1GALT1 and T antigen levels in BLCA (T) and adjacent normal (A) tissues. *p < 0.05; ***, *p* < 0.001. **E** T antigen levels in BLCA patient sera, by ELISA. *, *p* < 0.05. **F** mRNA expression of C1GALT1 in paired and unpaired BLCA and adjacent normal tissues in TCGA database. **, *p* < 0.01. **G** mRNA expression of C1GALT1 in BLCA patients from GSE27448 series. **, *p* < 0.01. **H, I** Overall (**H**) and disease-free survival (**I**) of dichotomized C1GALT1 mRNA expression in BLCA patients in TCGA database, using GEPIA platform. **J** Left: representative ESI-MS/MS spectra of O-glycan structures in HCV29 and YTS-1 cells. Right: relative abundance of T-antigen in normal epithelial bladder cells HUC-1 and HCV-29 vs. in bladder cancer cells KK47, T24 and YTS-1

Expression of C1GALT1 and its product T antigen (Fig. 1A) were evaluated by TMA analysis in 44 pairs of primary human BLCA tissue samples with matched adjacent noncancerous bladder tissues (Table S2). C1GALT1 expression was clearly higher in BLCA than in noncancerous cells (Fig. 1B), as were corresponding T antigen levels (Fig. 1C). Western and lectin blotting likewise revealed increased C1GALT1 expression and T antigen levels in BLCA tissues (Fig. 1D), and higher serum levels of T antigen in BLCA tissues were shown by lectin PNA-based ELISA (Fig. 1E). The Cancer Genome Atlas (TCGA) and Gene Expression Omnibus (GEO) databases indicate significantly higher C1GALT1 expression at mRNA level in BLCA relative to adjacent tissues (Fig. 1F, G, S1A). According to Kaplan-Meier curve analyses from TCGA database, overall survival prognosis was significantly worse for patients with high (vs. low) C1GALT1 expression (Fig. 1H), and high C1GALT1 expression in BLCA patients was correlated with poor disease-free survival (Fig. 1I). Similarly, high (vs. low) expression C1GALT1 or T antigen showed worse overall survival using TMA analyses (S1B, C).

In the present study, C1GALT1 expression was higher in invasive BLCA YTS-1 than in normal bladder HCV-29 cells (Fig. S1D). And O-glycan analysis showed higher T antigen levels in bladder cancer cells KK47, T24 and YTS-1, than in normal epithelial bladder cells HUC-1 and HCV-29 (Fig. 1J). These findings indicate that dysregulated expression of C1GALT1 and T antigen is characteristic of BLCA.

### Functional role of C1GALT1 in BLCA

**Fig. 2:**
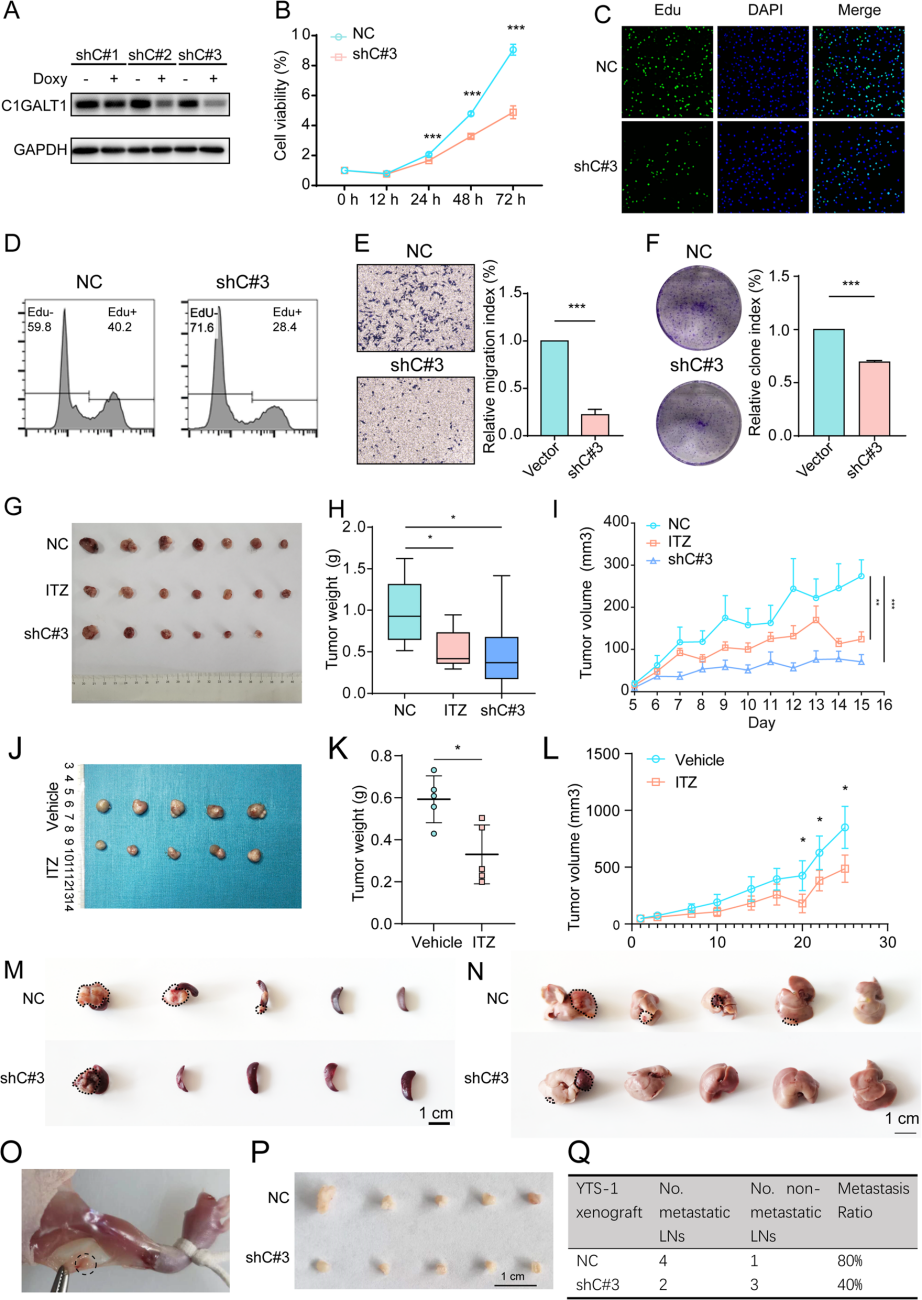
Effects of C1GALT1 on malignant behavior of BLCA cells. **A** C1GALT1 expression in C1GALT1-silenced YTS-1 cells (termed shC#1–3) with/without Doxy treatment. **B-D** Proliferation of C1GALT1-silenced YTS-1 vs. control cells by CCK8 (**B**) and Edu assay (**C, D**). ***, *p* < 0.001. **E, F** Migratory ability (**E**) and colony formation (**F**) of C1GALT1-silenced YTS-1 vs. control cells. ***, *p* < 0.001. **G-I** Tumor size (**G**), weight (**H**), and volume (**I**) for mice injected with C1GALT1-silenced, ITZ-treated, and control YTS-1 cells (*n* = 7). *, *p* < 0.05; **, *p* < 0.01; ***, *p* < 0.001. **J-L** Tumor size (**J**), weight (**K**) and volume (**L**) for PDXs with/without ITZ treatment. **M, N** Microphotographs of splenic (**M**) and liver metastatic tumors (**N**). **O** A popliteal lymphatic metastasis model was established by inoculating the foot pads of nude mice with YTS-1 cells. **P, Q** Size of LNs (**P**) and metastasis ratios (**Q**) of popliteal lymphatic metastasis mouse model

To investigate this topic, we silenced C1GALT1 in YTS-1, and established three stable transfectants (termed shC#1, shC#2, shC#3) that had low levels of C1GALT1 and T antigen response to Doxy treatment (Fig. 2A, S2A). The following experiments were performed using shC#3, which had the lowest C1GALT1 level. C1GALT1 silencing exhibited reduced migratory ability, colony formation, proliferation and resistance to doxorubicin, accompanied by decreased expression of FGFR3, one specific bladder protein marker [33] in YTS-1 and T24 cells (Figs. 2B-F, S2B-F). Treatment with ITZ, a pharmacological C1GALT1 inhibitor [34], significantly reduced C1GALT1 expression (Fig. S2G). Similarly to results from C1GALT1 silencing, ITZ treatment suppressed YTS-1 cell proliferation, colony formation, and migratory ability (Fig. S2H-K). Moreover, C1GALT1 overexpression in HCV29 cells resulted in the enhanced cell proliferation, colony formation, and migratory ability (Fig. S3A-D).

Tumor volume and weight in vivo were significantly reduced by ITZ treatment or C1GALT1 silencing (Fig. 2G-I). Immunohistochemical analyses revealed decreased T antigen and C1GALT1 expression and cell proliferation, and enhanced apoptosis, in ITZ-treated and C1GALT1-silenced tumor xenografts (Fig. S4A). We further established subcutaneous BLCA PDXs in mice. The tumors derived from PDXs with ITZ treatment regrew shortly compared to control group (Fig. 2J-L). Using PDXs model, we found that ITZ treatment inhibited C1GALT1 expression, enhanced apoptosis, and reduced proliferation (Fig. S4B).

In the trans-splenic metastasis model, C1GALT1 silencing significantly suppressed tumor metastasis to liver (Fig. 2M, N), enhanced apoptosis, and reduced proliferation (Fig. S5). In the popliteal lymphatic metastasis model (Fig. 2O), the volumes of lymphatic nodes were smaller in the C1GALT1 silencing group than which in control group (Fig. 2P). C1GALT1 silence suppressed the ability of the BLCA cells to metastasize to the LNs, as determined by the number of metastatic LNs and Haematoxylin & Eosin (H&E) staining (Fig. 2Q, S6A, B).

### Screening regulatory miRNAs of C1GALT1

**Fig. 3:**
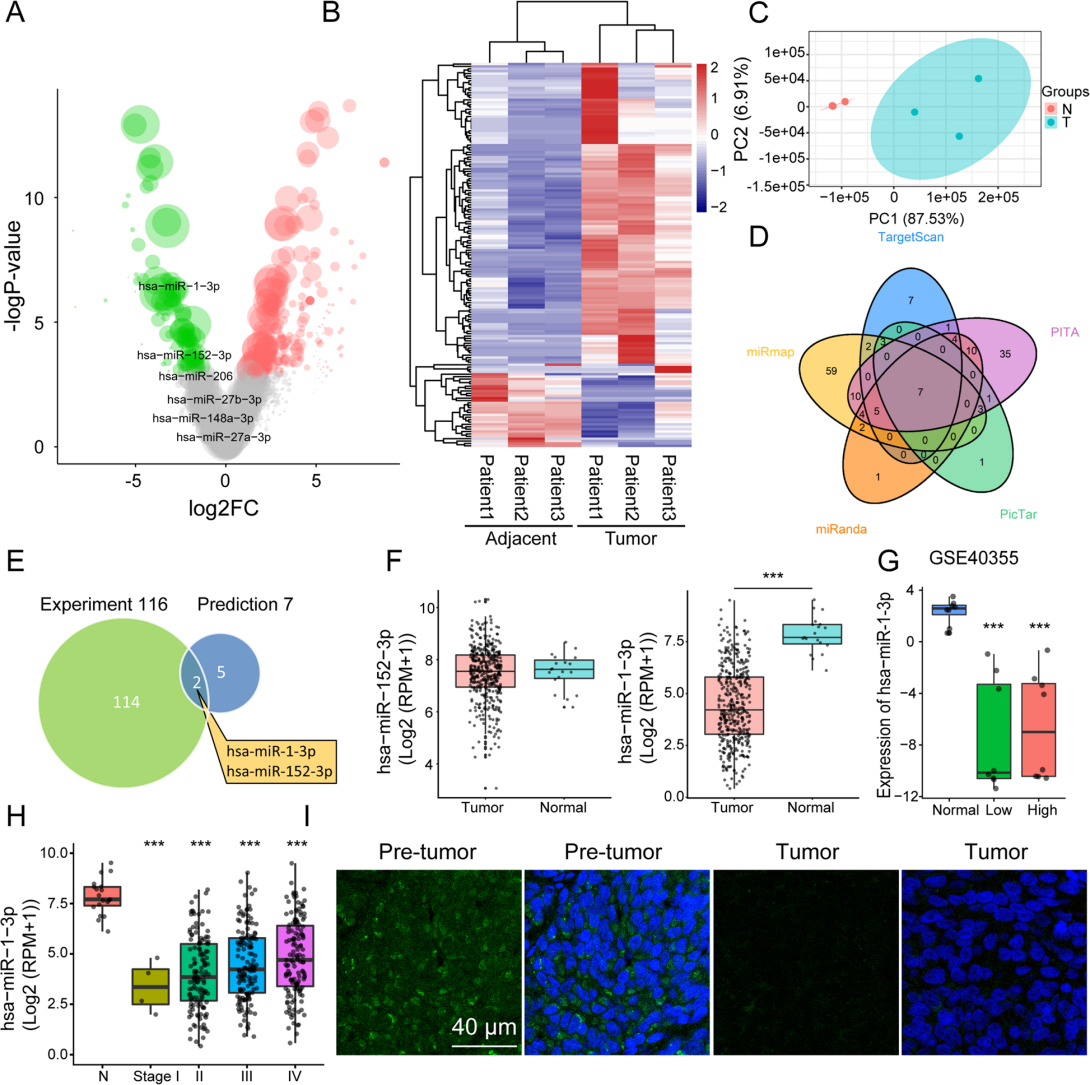
Identification of miRNAs in BLCA tissues by RNA-seq analysis. **A** Volcano plot of expression patterns of identified miRNAs in three pairs of BLCA tissues. -log10 (*p*-value) is plotted against log2 (fold change: BLCA vs. adjacent normal tissues) using cutoffs of fold change > 2, fold change < 0.5, and p-value < 0.05. Green, downregulation; red, upregulation. **B** Expression pattern of DemiRs identified in three pairs of BLCA tissues. Rows: miRNAs; columns: tissues. Red, upregulation; blue, downregulation. **C** PCA plot of DEmiRs. Green dots, BLCA tissues; red dots, adjacent normal tissues. **D** Venn diagram of predicted C1GALT1-targeting miRNAs by TargetScan, PITA, PicTar, miRanda, and miRmap. **E** Venn diagram of identified downregulated miRNAs and predicted C1GALT1-targeting miRNAs. **F** miR-1-3p and miR-152-3p expression in BLCA patients, from TCGA database. ***, *p* < 0.001. **G** miR-1-3p expression in low- and high-grade BLCA patients from GSE40355 series. ***, *p* < 0.001. **H** miR-1-3p expression in BLCA patients with stages I-IV, from TCGA database. ***, *p* < 0.001. **I** FISH analysis of miR-1-3p in BLCA and adjacent normal tissues. Nuclei were stained with DAPI, and miR-1-3p was stained with FITC-labeled probe

Regulatory functions of miRNAs on C1GALT1 in BLCA tissues were investigated by miRNA profiling. 1362 miRNAs were profiled in three pairs of BLCA tissues, and 161 differentially expressed miRNAs (DEmiRs) were identified (Table S3). Expression patterns of DEmiRs in BLCA vs. adjacent normal tissues were presented by volcano plot (Fig. 3A). 129 miRNAs were upregulated and 32 miRNAs were downregulated in BLCA tissues (Fig. 3B). Principal component analysis (PCA) revealed clear separation of DEmiRs in BLCA vs. normal tissues (Fig. 3C). Seven miRNAs were predicted to target C1GALT1 according to public databases (Fig. 3D). To assess negative regulatory role of miRNAs, miR-1-3p and miR-152-3p were filtered based on overlap between downregulated miRNAs and predicted target miRNAs of C1GALT1 (Fig. 3E). miRNA sequencing datasets in TCGA indicated significant decrease of miR-1-3p, but not miR-152-3p, in BLCA tissues (Fig. 3F). Reduced miR-1-3p expression was consistently observed in low-grade and high-grade BLCA, and in stages I-IV (Fig. 3G-H), and low expression was confirmed by FISH assay (Fig. 3I). These findings, taken together, indicate targeting of C1GALT1 by miR-1-3p in BLCA.

### Suppressing effect of miR-1-3p on C1GALT1

**Fig. 4:**
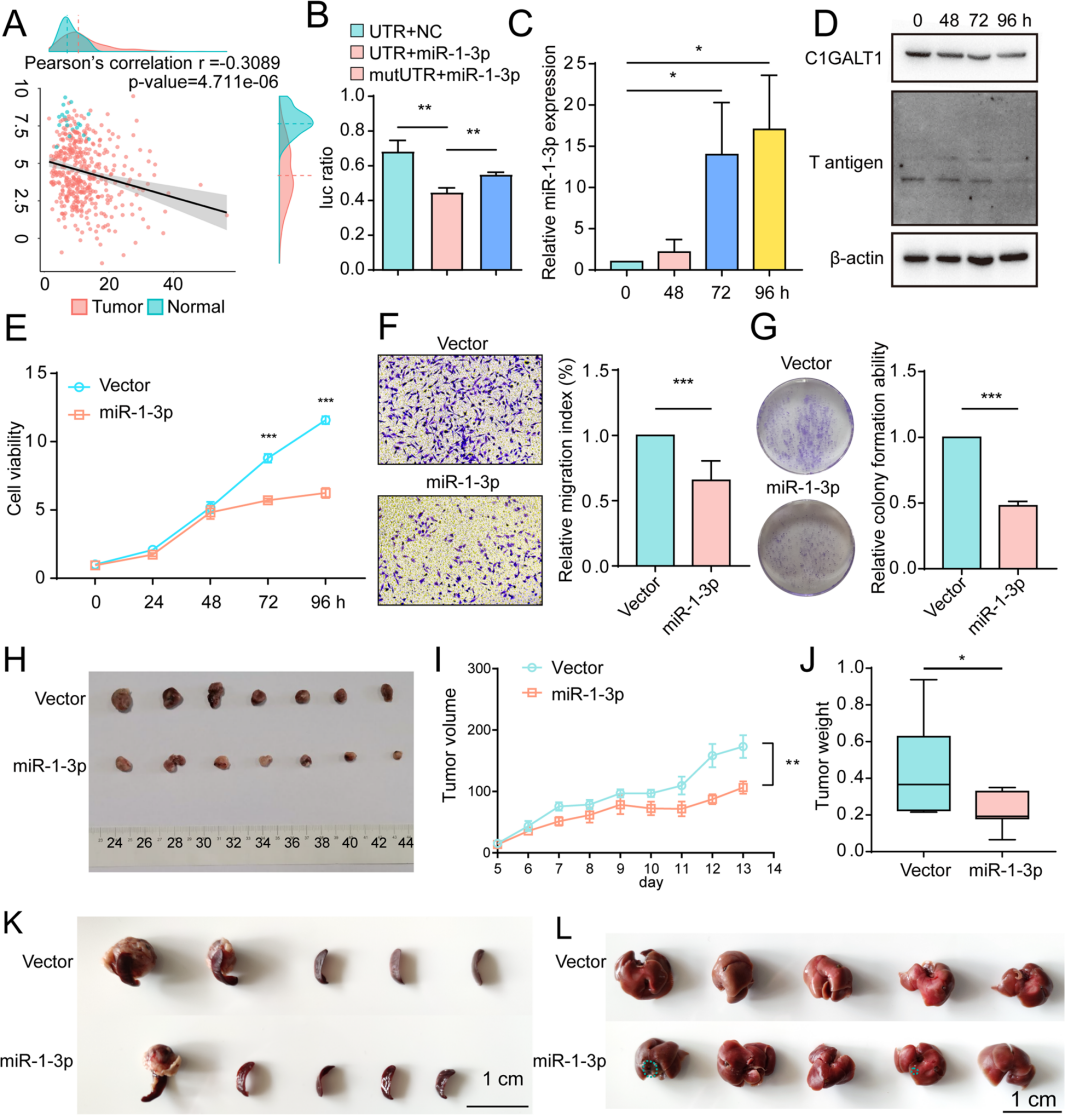
Effects of miR-1-3p on malignant behavior of BLCA cells. **A** Correlation analysis of miR-1-3p and C1GALT1 mRNA expression in BLCA patients, from TCGA database. **B** Confirmation of miR-1-3p-binding sites on 3′-UTR of C1GALT1 detected by luciferase reporter assay. Control miRNA or miR-1-3p mimics were co-transfected with luciferase reporters containing wild-type or mutated 3′-UTRs. **, *p* < 0.01. **C, D** miR-1-3p (**C**), C1GALT1 expression, and T antigen levels (**D**) in miR-1-3p-overexpressing YTS-1 cells with Doxy treatment. *, *p* < 0.05. **E-G** Proliferation (**E**), migratory ability (**F**), and colony formation (**G**) of miR-1-3p-overexpressing YTS-1 vs. control cells. ***, *p* < 0.001. **H-J** Tumor size (**H**), volume (**I**), and weight (**J**) at wk. 3 after s.c. injection of miR-1-3p-overexpressing YTS-1 cells (*n* = 7). *, *p* < 0.05; **, *p* < 0.01. **K, L** Microphotographs of splenic (**K**) and liver metastatic tumors (**L**)

Interaction between C1GALT1 mRNA and miR-1-3p in BLCA was examined in view of the above findings. According to TCGA database, C1GALT1 expression is negatively correlated with miR-1-3p expression in BLCA patients and healthy controls (Fig. 4A). In luciferase reporter assay, miR-1-3p bound directly to 3′-UTR of C1GALT1, but not of mutated 3′-UTR (Fig. 4B). miR-1-3p-overexpressing YTS-1 and T24 cells showed significant reduction of C1GALT1 and T antigen expression (Fig. 4C-D, S7A), cell proliferation, migratory ability, colony formation, and doxorubicin resistance (Figs. 4E-G, S7B-D). miR-1-3p mimics similarly inhibited C1GALT1 expression, cell proliferation, and migratory ability, whereas miR-1-3p inhibitor enhanced these processes (Fig. S7E-G).

In vivo studies revealed significant reduction of tumor volume and weight in miR-1-3p-overexpressing cells (Fig. 4H-J). Immunohistochemical analysis of these cells showed decreased C1GALT1 and T antigen expression and cell proliferation, and increased apoptosis (Fig. S7H). In the trans-splenic metastasis model, miR-1-3p overexpression significantly reduced tumor metastasis to liver and cell proliferation, and increased apoptosis (Fig. 4K, L, S8).

### Screening regulatory circRNAs of miR-1-3p

**Fig. 5:**
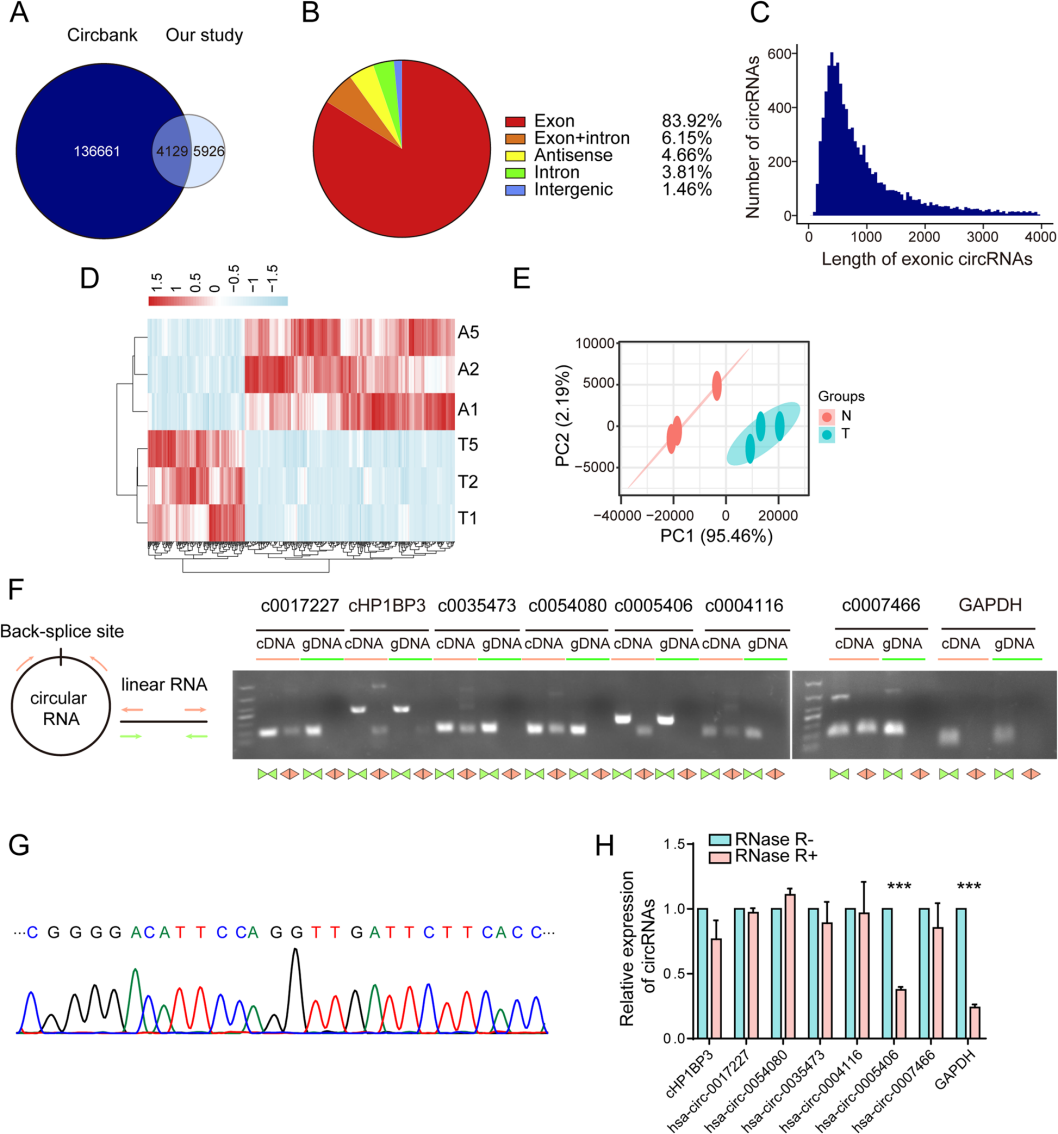
Identification of circRNAs in BLCA tissues by RNA-seq analysis. **A** Overlap of circRNAs identified in this study and in circBank. **B** Genomic origin or identified circRNAs in BLCA tissues. **C** Length distribution of exonic circRNAs. **D** Heatmap of DEcircRs in three pairs of BLCA tissues. Rows: circRNAs; columns: tissues. Red, upregulation; blue, downregulation. **E** PCA plot of DEcircRs. Green dots, BLCA tissues; red dots, adjacent normal tissues. **F** Representative PCR confirmation of predicted circRNA backsplice junctions using divergent primers and host genes for circRNA, and convergent primers for cDNA and genomic DNA. **G** Confirmation of circRNA (cHP1BP3) backsplice junctions by Sanger sequencing. **H** RNase R resistance of circRNAs and GAPDH mRNA. ***, *p* < 0.001

CircRNAs have been shown to act as miRNA “sponges”, and to play essential roles at the transcriptional level. Association of circRNAs with miR-1-3p was investigated by characterizing circRNA transcripts through RNA-seq analysis of rRNA-depleted and RNase-treated RNA from pairs of BLCA tissues as described above. We identified 10,055 circRNAs in these tissues, including 4129 that overlapped with circBank [35] and 5926 novel circRNAs (Fig. 5A). Of these, 8438 circRNAs (83.9%) consisted of protein coding exons from 3519 host genes, and the rest consisted of exon-intron, intron, intergenic, or antisense (Fig. 5B). Most of the exonic circRNAs had lengths < 1000 nt (Fig. 5C). Identified circRNAs were quantified, and 414 were found to be differentially expressed in BLCA tissues relative to matched adjacent normal tissues (Table S4). Of these differentially expressed circRNAs (termed DEcircRs), 283 were upregulated and 131 were downregulated in BLCA tissues (Fig. 5D). PCA of DEcircRs demonstrated clear distinction of those in BLCA vs. adjacent normal tissues (Fig. 5E). Of the 414 DEcircRs, 349 were exonic, of which 244 were documented in circBank. For confirmation of RNA-seq findings, we examined the top four upregulated and bottom three downregulated DEcircRs. Head-to-tail splicing was assayed by RT-qPCR, with divergent primers and Sanger sequencing (Figs. 5F-G, S9A-B). Head-to-tail junctions of the seven DEcircRs as above were all confirmed, demonstrating high accuracy of our circRNA profiling. We examined sensitivity of the seven DEcircRs to RNase R digestion, to rule out possible production of head-to-tail splicing by trans-splicing, genomic rearrangements, or PCR artefacts. The DEcircRs all showed greater resistance to RNase R than that of GAPDH control (Fig. 5H). In conclusion, characterized circRNAs were consisted mainly of exons, and seven selected DEcircRs were confirmed as having head-to-tail junctions.

### Sponage role of cHP1BP3 on miR-1-3p

**Fig. 6:**
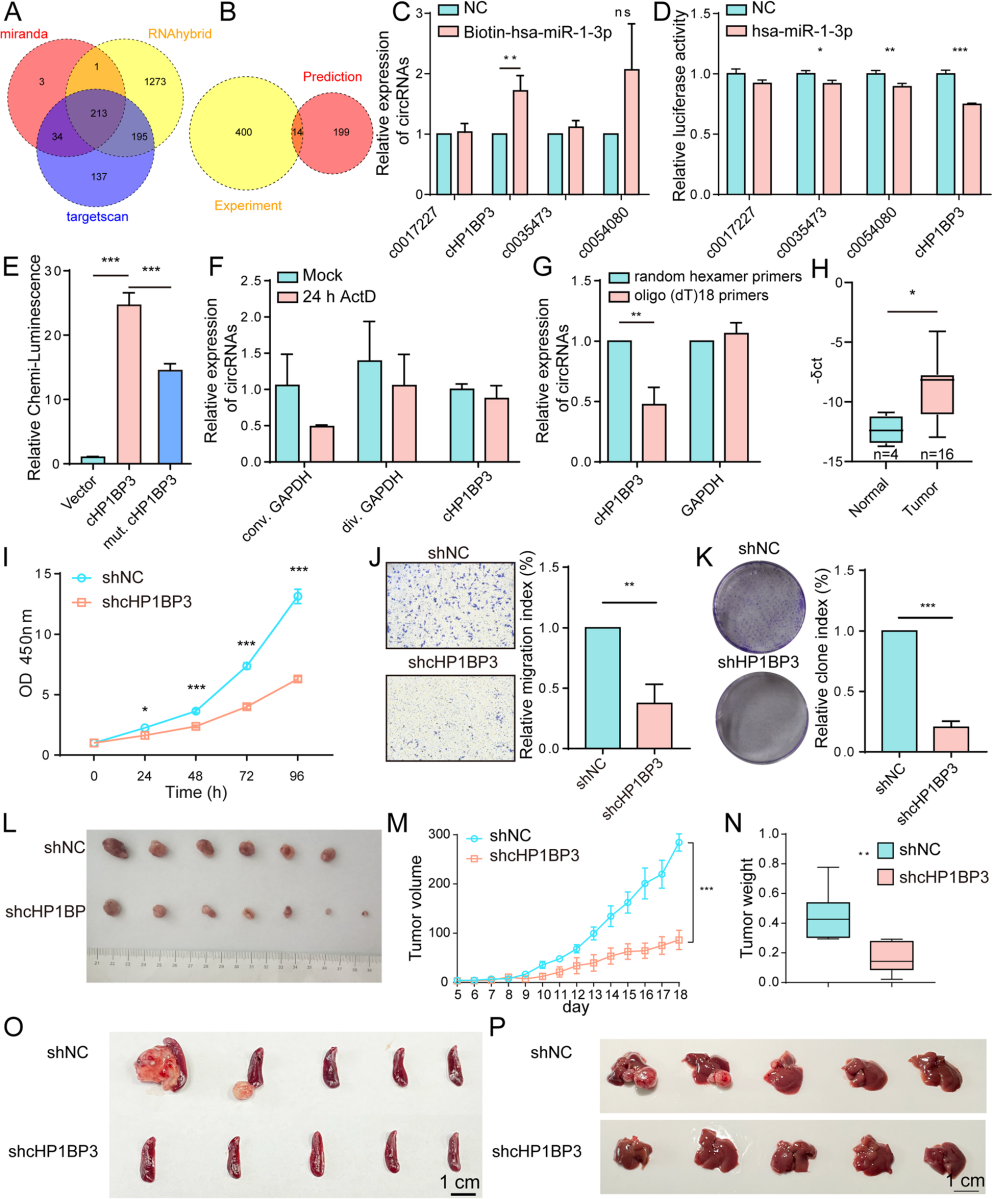
Effects of cHP1BP3 on malignant behavior of BLCA cells. **A** Venn diagram of predicted circRNAs acting as miR-1-3p sponges, from miRanda, RNAhybrid, and TargetScan databases. **B** Venn diagram of identified upregulated circRNAs and predicted circRNAs acting as miR-1-3p sponges. **C** RT-qPCR analysis of circRNA levels in streptavidin-captured fractions from YTS-1 cell lysates following transfection with 3′-end biotinylated miR-1-3p or control RNA (NC). **, *p* < 0.01. **D** Confirmation by luciferase reporter assay of miR-1-3p-binding sites on circRNAs. Control miRNA or miR-1-3p mimics were co-transfected with luciferase reporters containing predicted miR-1-3p binding sites. *, *p* < 0.05; **, *p* < 0.01; ***, *p* < 0.001. **E** Luciferase activity of luciferase-cHP1BP3 or its mutant in HEK293 cells following co-transfection with miR-1-3p. ***, *p* < 0.001. **F** Stability of cHP1BP3 following 24 h transcription blocking by actinomycin D (ActD) treatment, by RT-qPCR analysis. **G** Expression of cHP1BP3 amplified with random hexamer or oligo (dT)18 primers, by RT-qPCR. **, *p* < 0.01. **H** cHP1BP3 expression in BLCA and adjacent normal tissues. **I-K** Proliferation (**I**), migratory ability (**J**), and colony formation (**K**) of cHP1BP3-silenced YTS-1 vs. control cells. *, *p* < 0.05; **, *p* < 0.01; ***, *p* < 0.001. **L-N** Tumor size (**L**), volume (**M**), and weight (**N**) at wk. 3 after s.c. injection of cHP1PB3-silenced YTS-1 cells (*n* = 7). **, *p* < 0.01; ***, *p* < 0.001. **O, P** Microphotographs of splenic (**O**) and liver metastatic tumors (**P**)

Potential regulatory relationships between miR-1-3p and circRNAs were evaluated using the three public databases. 213 circRNAs were predicted to target hsa-miR-1-3p based on our results (Fig. 6A). In view of the miRNA sponge function of circRNA, 14 circRNAs were filtered by overlap between DEcircRs and predicted target circRNAs of miR-1-3p (Fig. 6B). Among these, the top four upregulated circRNAs were selected for examining interaction with miR-1-3p. Biotin-labeled miR-1-3p was introduced into YTS-1 cells, and interactions were evaluated by RNA pulldown, which revealed strong binding of circRNA cHP1BP3 to miR-1-3p (Fig. 6C). Luciferase reporter assay indicated direct binding of miR-1-3p to predicted cHP1BP3 binding sites (Fig. 6D), other than mutated sites (Figs. 6E, S10A). cHP1BP3 was highly stable in response to transcriptional blockage, other than linear counterpart GAPDH (Fig. 6F). cHP1BP3 was significantly downregulated relative to oligo (dT)_18_ primers when random hexamer primers were used for reverse transcription experiments (Fig. 6G), indicating the circular nature of cHP1BP3. FISH assay showed that cHP1BP3 was located mainly in cytoplasm, and significantly enhanced in BLCA vs. adjacent normal tissues (Fig. S10B). cHP1BP3 level was negatively correlated with linear counterpart (Fig. S10C), indicating that its higher expression in BLCA reflected its functionality, and was not simply a by-product of splicing. Moreover, cHP1BP3 expression was significantly increased in BLCA tissues compared to normal tissues (Fig. 6H).

cHP1BP3 was silenced in YTS-1 and T24 cells to further elucidate its physiological functions. cHP1BP3 expression was significantly reduced by Doxy treatment using divergent primers (Fig. S11A), and cHP1BP3 silencing suppressed T antigen and C1GALT1 expression in YTS-1 and T24 cells (Fig. S11B, C). cHP1BP3 silencing also reduced cell proliferation, migratory ability, colony formation, and doxorubicin resistance relative to vehicle control (Figs. 6I-K, S11D-F), and suppressed tumor volume and weight in vivo (Fig. 6L-N). Immunohistochemical analysis confirmed that cHP1BP3 silencing suppressed T antigen and C1GALT1 expression, leading to reduced cell proliferation and increased apoptosis (Fig. S11G). In the trans-splenic metastasis model, cHP1BP3 silencing significantly suppressed tumor metastasis to liver (Fig. 6O, P), enhanced apoptosis, and reduced proliferation (Fig. S12), similarly to miR-1-3p overexpression (Fig. 4).

### Target proteins of C1GALT1

**Fig. 7:**
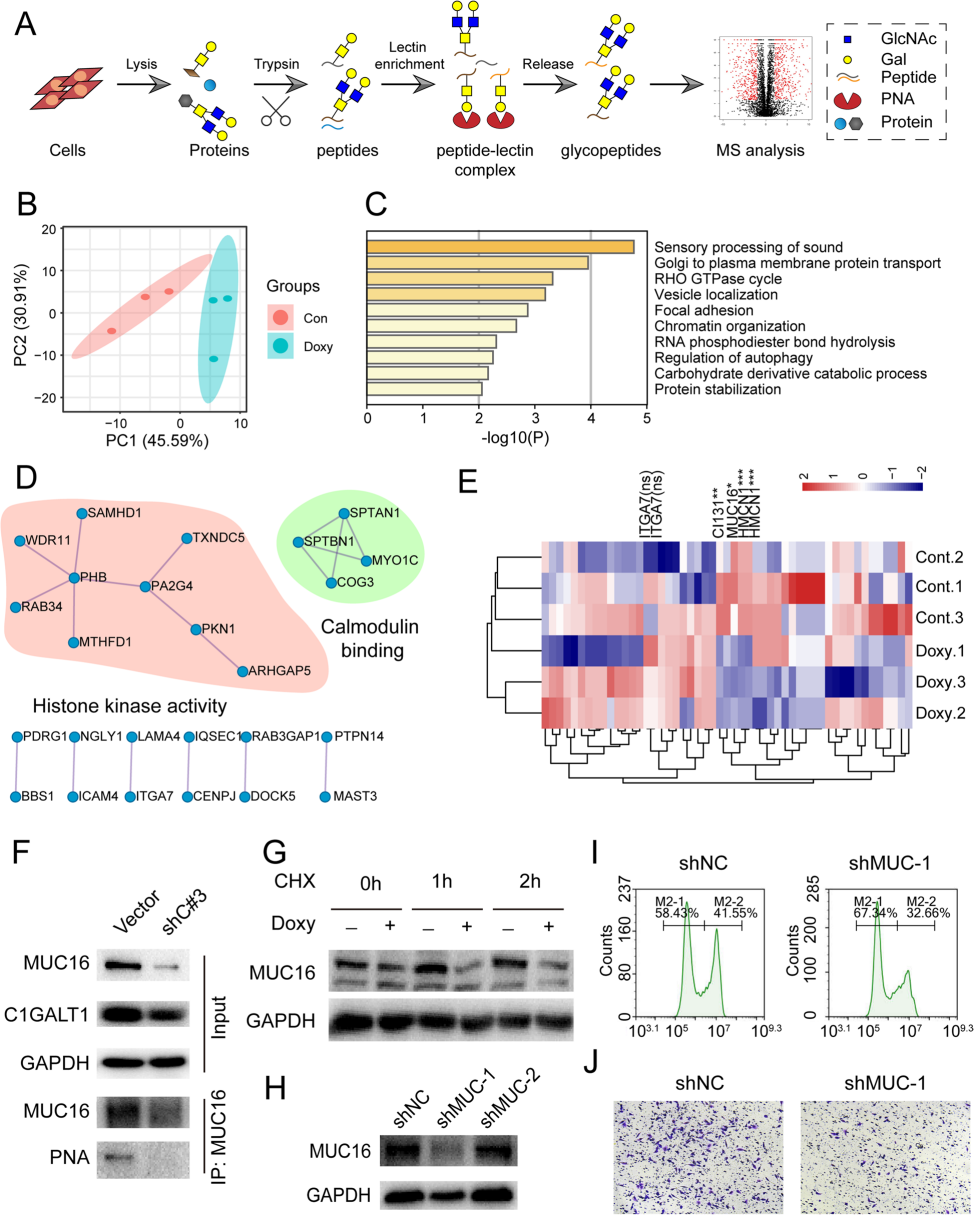
Identification of T antigen-bearing glycoproteins. **A** Glycoproteomic analysis by combination of PNA enrichment and LC-MS/MS (schematic). **B** PCA plot of identified glycoproteins. Green dots, C1GALT1-silenced YTS-1 cells; red dots, control cells. **C** GO enrichment analysis of identified glycoproteins. **D** Protein-protein interaction network of glycoproteins, by Cytoscape software program. **E** Heatmap showing expression patterns of glycoproteins identified in C1GALT1-silenced and parental YTS-1 cells. Red, upregulation; blue, downregulation. *, *p* < 0.05; **, *p* < 0.01; ***, *p* < 0.001; ns, not significant. **F** MUC16 expression and T antigen levels on MUC16 in control and C1GALT1-silenced YTS-1 cells. **G** MUC16 expression in control and C1GALT1-silenced YTS-1 cells treated with CHX. **H** MUC16 expression in MUC16-silenced YTS-1 cells by siRNA. **I, J** Proliferation (**I**) and migratory ability (**J**) of MUC16-silenced YTS-1 vs. control cells

C1GALT1 controls the essential step of O-glycosylation elongation, and strongly affects target protein function. Target O-glycosylated proteins of C1GALT1 involved in BLCA were examined by glycoproteomic analysis in combination with PNA enrichment and triplicate LC-MS/MS (Fig. 7A). 117 glycopeptides were identified in C1GALT1-silenced and parental YTS-1 cells, covering 67 glycoproteins (Table S5). PCA showed clear distinction of these glycoproteins in C1GALT1-silenced vs. parental YTS-1 (Fig. 7B). Gene Ontology (GO) and KEGG enrichment analyses revealed enrichment of proteins involved in protein transport from Golgi to plasma membrane, Rho GTPase cycle, and vesicle localization (Fig. 7C). Protein-protein interaction network analysis indicated that target proteins formed clusters with enriched histone kinase activity and calmodulin binding (Fig. 7D). Complete hierarchical clustering and visualization (heatmap) showed clustering of the identified glycoproteins (Fig. 7E), and their expression patterns in C1GALT1-silenced and parental YTS-1 cells. Quantification of the glycopeptides showed that three (including MUC16 and HMCN1) were differentially expressed (Fig. 7E). Consistently, immunoprecipitation analysis indicated that MUC16 was decorated with T antigen and MUC16 expression was decreased in C1GALT1 silenced YTS-1 cells (Fig. 7F). C1GALT1 silencing facilitated the degradation of MUC16 in YTS-1 cells treated with cycloheximide, the inhibitor of protein synthesis (Fig. 7G). MUC16 silencing by siRNA (Fig. 7H) suppressed the cell proliferation and migratory ability of YTS-1 cells (Fig. 7I, J). Collectively, these results indicated that C1GALT1 silencing suppressed migratory and proliferation of BLCA cells by modifying target glycoproteins including MUC16.

## Discussion

Truncated O-glycans and responsible enzyme C1GALT1 play important roles in a variety of physiological and pathological processes, including cancer development and progression [36, 37]. C1GALT1 overexpression has been reported for various cancers [18, 38–40]. Recent studies demonstrated that T antigen, the product of C1GALT1 activity, was present in high-grade BLCA but not in normal urothelium [41]. BLCA cells under hypoxic conditions showed striking upregulation of C1GALT1 and associated downregulation of C2GnT, the enzyme that catalyzes O-glycan core 2 formation with T antigen as substrate; this observation may account for T antigen accumulation in BLCA [41]. Consistant with other reports, we found that T antigen and its responsible enzyme C1GALT1 were up-regulated in BLCA, and C1GALT1 expression was positively correlated with malignant phenotypes by modulating C1GALT1 expression in BLCA cells. Notably, the treatment of ITZ, as one antifungal medication, could down-regulate the C1GALT1 expression, and suppress BLCA tumor growth and migratory ability in cell- and patient-derived xenografts, indicating the therapeutic potential of ITZ in BLCA.

In recent studies, C1GALT1 has been shown to be negatively modulated, similarly, by miR-181d-5p in lung adenocarcinoma(19), miR-152 in gastric cancer(16), and miR-124-3p in aging colon (20). In this study, C1GALT1 was revealed to be regulated by miR-1-3p, not by other miRNAs. These findings indicate that miRNAs targeting C1GALT1 have differential functions depending on cancer type. In a study of prostate cancer, miR-1-3p showed downregulation, and its ectopic expression suppressed cell growth and cell cycle progression in vitro and in vivo through targeting of 3′-UTRs of two key cell cycle genes, E2F5 and PFTK1 [42]. In non-small cell lung cancer, levels of miR-1 were low, and its overexpression inhibited tumor growth and angiogenesis by targeting Mpl gene [43], similarly to our findings.

**Fig. 8:**
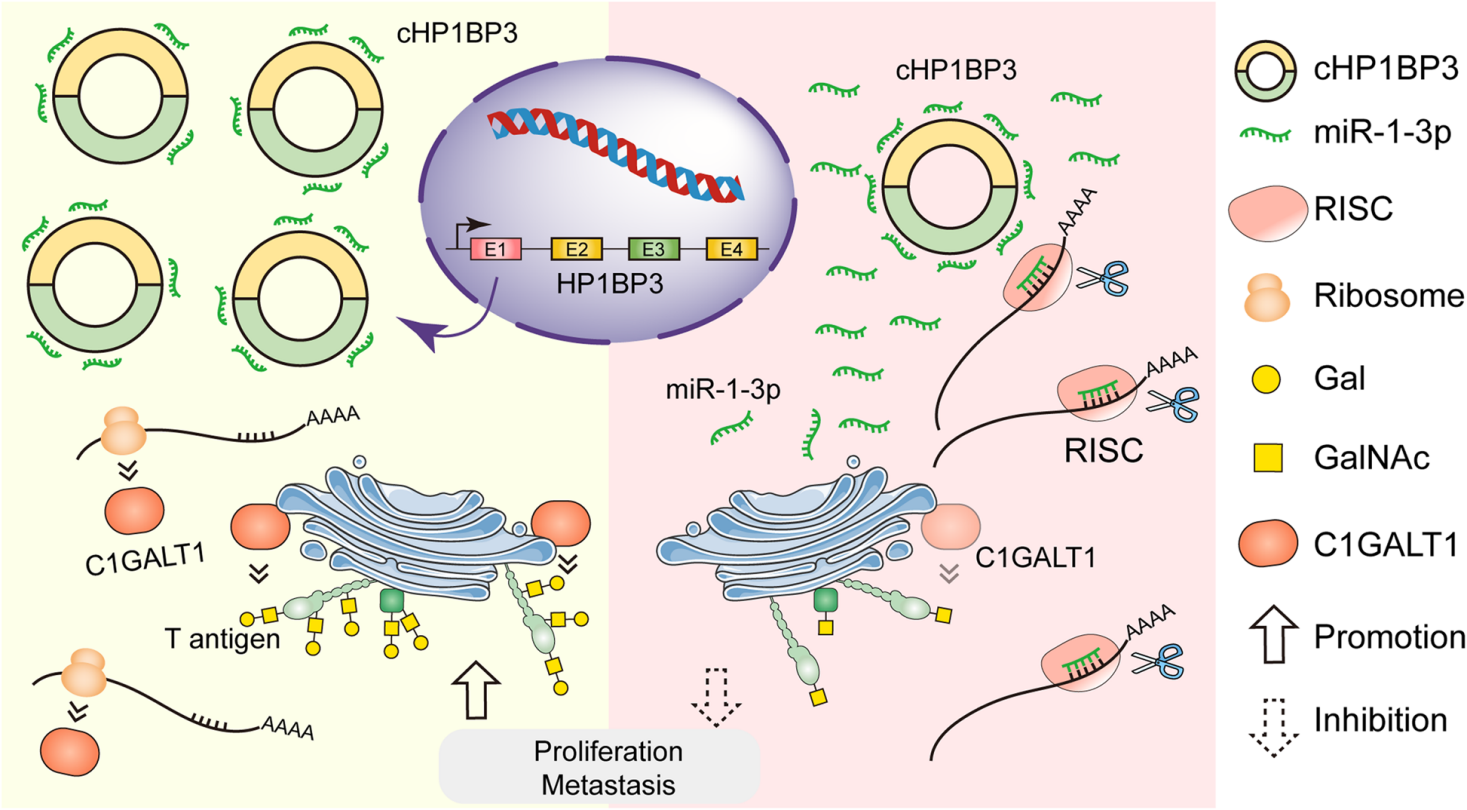
Proposed prometastatic function of C1GALT1 and its modulation by cHP1BP3/ miR-1-3p axis (schematic)

circRNAs have been shown to act as miRNA sponges, participate in target gene splicing, transcribe genes into proteins, and interact with RNA-binding proteins. We therefore examined the regulatory role of circRNA in abnormal C1GALT1 expression in BLCA. cHP1BP3 comprising several exons was identified as a significantly upregulated circRNA in BLCA, and was predicted by bioinformatic analysis to bind miR-1-3p. cHP1BP3 functions as a competing endogenous RNA (ceRNA), and was shown by biotinylated miRNA pulldown and luciferase reporter assays to bind competitively to miR-1-3p. In rescue experiments, cHP1BP3 abolished the endogenous suppressive effect of miR-1-3p on target C1GALT1. These observations, in combination with our identification of C1GALT1 as the direct target of miR-1-3p, indicate that C1GALT1 is regulated via cHP1BP3/ miR-1-3p axis in BLCA (Fig. 8).

C1GALT1 is able to modify O-glycans on target proteins and regulate their physiological functions. We performed PNA enrichment of T antigen-bearing glycoproteins followed by MS analysis, and observed that integrin α7 was bearing T antigen. Previous studies have demonstrated similar O-glycosylation changes on integrin β1, αv, and α5 subunits. C1GALT1 affects cell migratory ability and invasiveness by modifying O-glycans on integrin α5 [44] or β1 [39], and modulating their downstream signaling. C1GALT1 knockdown enhanced radiosensitivity of esophageal cancer cells, and suppressed irradiation-enhanced migration and invasion by modifying glycosylation of integrin β1 [45]. MUC16 has also been identified as a C1GALT1 target glycoprotein in BLCA. Pancreas-specific C1GALT1 knockdown resulted in early onset of tumors and metastatic lesions, possibly because of dysregulated MUC16 O-glycan modification [16]. Deletion of MUC16 decreases tumor cell migration in pancreatic ductal adenocarcinoma cells [46], which is in line with the decrease in proliferation and migratory ability in MUC16-silenced YTS-1 cells. MUC16 bearing truncated O-glycans exacerbates malignancy by FAK activation through interactions with integrins on cell membranes [46], indicating that O-glycans might regulate the localization and turnover of target proteins and be involved in cancer progression.

## Conclusions

In conclusion, our findings indicate that C1GALT1 is modulated by cHP1BP3/ miR-1-3p axis and upregulated in BLCA. C1GALT1 silencing suppressed migratory ability and proliferation of BLCA cells in vitro and in vivo by modifying target glycoproteins. Notably, C1GALT1 is revealed to be regulated via circRNA/ miRNA axis in BLCA, which might provide theoretical basis for the diagnosis and therapy of BLCA.

## Supplementary Information

**Additional file 1: Table S1.** DNA oligo sequences.

**Additional file 2: Table S2.** Patient information for analysis of C1GALT1 and T antigen expression in tumor and adjacent tissues.

**Additional file 3: Table S3.** Differentially expressed miRNAs in tumor and adjacent tissues.

**Additional file 4: Table S4.** Differentially expressed circRNAs in tumor and adjacent tissues.

**Additional file 5: Table S5.** Proteins modified with T antigen identified in control and C1GALT1 silenced YTS-1 cells.

**Additional file 6: Fig. S1.** Expression of C1GALT1 in BLCA cells and tissues. A C1GALT1 mRNA expression in various stages of BLCA patients, in TCGA database. B-C Overall survival of dichotomized C1GALT1 (B) and T antigen (C) expression in BLCA patients using TMA. D C1GALT1 expression and T antigen levels in various BLCA (5637, RT4, KK47, J82, T24, YTS-1) and normal uroepithelial (HCV29, HUC-1) cell lines. **Fig. S2.** Effects of C1GALT1 silencing on malignant behavior of YTS-1 and T24 cells. (A) T antigen levels in C1GALT1-silenced YTS-1 cells by flow cytometry. (B) FGFR3 expression in C1GALT1-silenced YTS-1 cells. (C) Doxorubicin resistance of control and C1GALT1-silenced YTS-1 cells. (D) C1GALT1 expression in C1GALT1 silenced T24 cells. (E, F) Proliferation (E) and migratory ability (F) of C1GALT1-silenced YTS-1 cells. (G) C1GALT1 expression in ITZ-treated YTS-1 cells. (H-K) Proliferation (H), colony formation (I), migratory ability (J), and doxorubicin resistance (K) of ITZ-treated YTS-1 vs. control cells. **Fig. S3.** Effects of C1GALT1-overexpressing on malignant behavior of HCV29 cells. (A) C1GALT1 expression in C1GALT1-overexpressing HCV29 cells. (B-D) Proliferation (B), colony formation (C) and migratory ability (D) of C1GALT1-overexpressing HCV29 cells. **Fig. S4.** Immunohistochemical analysis of tumors of mice model. (A) Immunohistochemical analysis of Ki67, TUNEL, C1GALT1, and T antigen in tumors of mouse injected with C1GALT1-silenced, ITZ-treated, and control YTS-1 cells. (B) Immunohistochemistry analysis of Ki67, TUNEL and C1GALT1 in PDXs with or without ITZ treatment. **Fig. S5.** Immunohistochemical analysis of C1GALT1, T antigen, Ki67, and TUNEL in tumors of trans-splenic metastasis model mice injected with control and C1GALT1-silenced YTS-1 cells. **Fig. S6.** H&E staining and immunohistochemistry analysis of LNs of popliteal lymphatic metastasis mouse model. (A, B) H&E staining (A) and immunohistochemistry analysis (B) of LNs of popliteal lymphatic metastasis model mouse injected with C1GALT1-silenced and control YTS-1 cells into footpads. **Fig. S7.** Effects of miR-1-3p overexpressing on malignant behavior of BLCA YTS-1 cells. (A) C1GALT1 expression in miR-1-3p-overexpressing T24 cells. (B, C) Proliferation (B) and migratory ability (C) of miR-1-3p-overexpressing T24 cells. (D) Doxorubicin resistance of control and miR-1-3p-overexpressing YTS-1 cells. (E) C1GALT1 and T antigen levels of YTS-1 cells transfected with miR-1-3p mimic or inhibitors. (F, G) Proliferation (F) and migratory ability (G) of YTS-1 cells transfected with miR-1-3p mimic or inhibitor, in comparison with control cells. (H) Immunohistochemical analysis of Ki67, TUNEL, C1GALT1, and T antigen in tumors of mouse injected with miR-1-3p-overexpresssing and control YTS-1 cells. **Fig. S8.** Immunohistochemical analysis of C1GALT1, T antigen, Ki67, and TUNEL in tumors of trans-splenic metastasis model mice injected with control and miR-1-3p-overexpressing YTS-1 cells. **Fig. S9.** Confirmation of circRNA backsplice junctions by Sanger sequencing. (A) Sequences of backsplice junctions of selected circRNAs. Arrows: backsplice sites. (B) Production of cHP1BP3 (schematic). **Fig. S10.** Expression of circular cHP1BP3 and its linear counterpart in BLCA cells. (A) Sequence of mutated cHP1BP3 binding sites targeted by miR-1-3p. (B) RNA FISH for cHP1BP3 in BLCA and adjacent normal tissues. Nuclei were stained with DAPI, and miR-1-3p was FITC-labeled. (C) Expression of linear counterpart of cHP1BP3 in BLCA patients, in TCGA database. **Fig. S11.** Effects of cHP1BP3 silencing on malignant behavior of HCV29 and T24 cells. (A-C) Expression of cHP1BP3 (A), C1GALT1, and T antigen (B, C) in cHP1BP3-silenced YTS-1 and T24 cells. (D) Doxorubicin resistance of control and cHP1BP3-silenced YTS-1 cells. (E, F) Proliferation (E) and migratory ability (F) of cHP1BP3-silenced T24 cells. (G) Immunohistochemical analysis of Ki67, TUNEL, C1GALT1, and T antigen in tumors of mouse injected with control and cHP1BP3-silenced YTS-1 cells. **Fig. S12.** Immunohistochemical analysis of C1GALT1, T antigen, Ki67, and TUNEL in tumors of trans-splenic metastasis model mice injected with control and cHP1BP3-silenced YTS-1 cells.

## Data Availability

The data supporting the conclusions of this article have been given in this article and its additional files.
