# Supplementary material for: Dysregulation and prometastatic function of glycosyltransferase C1GALT1 modulated by cHP1BP3/ miR-1-3p axis in bladder cancer
Source: J Exp Clin Cancer Res. 2022 Jul 21;41:228. doi: 10.1186/s13046-022-02438-7 (PMC9306173; doi:10.1186/s13046-022-02438-7)

**Supplementary Information**

The dysregulation and prometastatic function of C1GALT1 modulated by cHP1BP3/miR-1-3p axis in bladder cancer

Zengqi Tan^1,#^, Yazhuo Jiang^2,#^, Liang Liang^3#^, Jinpeng Wu^4^, Lin Cao^1^, Xiaoman Zhou^4^, Zhihui Song^4^, Zhenyu Ye^4^, Ziyan Zhao^4^, Hui Feng^4^, Zewen Dong^4^, Shuai Lin^5^, Zhangjian Zhou^5^, Yili Wang^6^, Xiang Li^1*^, Feng Guan^1,4*^

^1^ Institute of Hematology, Provincial Key Laboratory of Biotechnology, School of Medicine, Northwest University, Xi'an, 710069, P.R. China

^2^ Department of Urology, The Third Affiliated Hospital of Xi'an Jiaotong University, Xi'an, 710068, P.R. China

^3^ Department of Urology, The First Affiliated Hospital of Xi'an Jiaotong University, Xi'an, 710061, P.R. China

^4^ Key Laboratory of Resource Biology and Biotechnology in Western China, Ministry of Education, Provincial Key Laboratory of Biotechnology, College of Life Sciences, Northwest University, Xi'an, 710069, P.R. China

^5^ Department of Oncology, The Second Affiliated Hospital of Xi’an Jiaotong University, Xi'an, 710061, P.R. China

^6^ Institute for Cancer Research, School of Basic Medical Science, Health Science Center of Xi’an Jiaotong University, Xi’an, 710061, P.R. China

# These authors contributed equally to the study.

Fig. S1. Expression of C1GALT1 in BLCA cells and tissues

**(A)** C1GALT1 mRNA expression in various stages of BLCA patients, in TCGA database. **(B-C)** Overall survival of dichotomized C1GALT1 (B) and T antigen (C) expression in BLCA patients using TMA. **(D)** C1GALT1 expression and T antigen levels in various BLCA (5637, RT4, KK47, J82, T24, YTS-1) and normal uroepithelial (HCV29, HUC-1) cell lines.


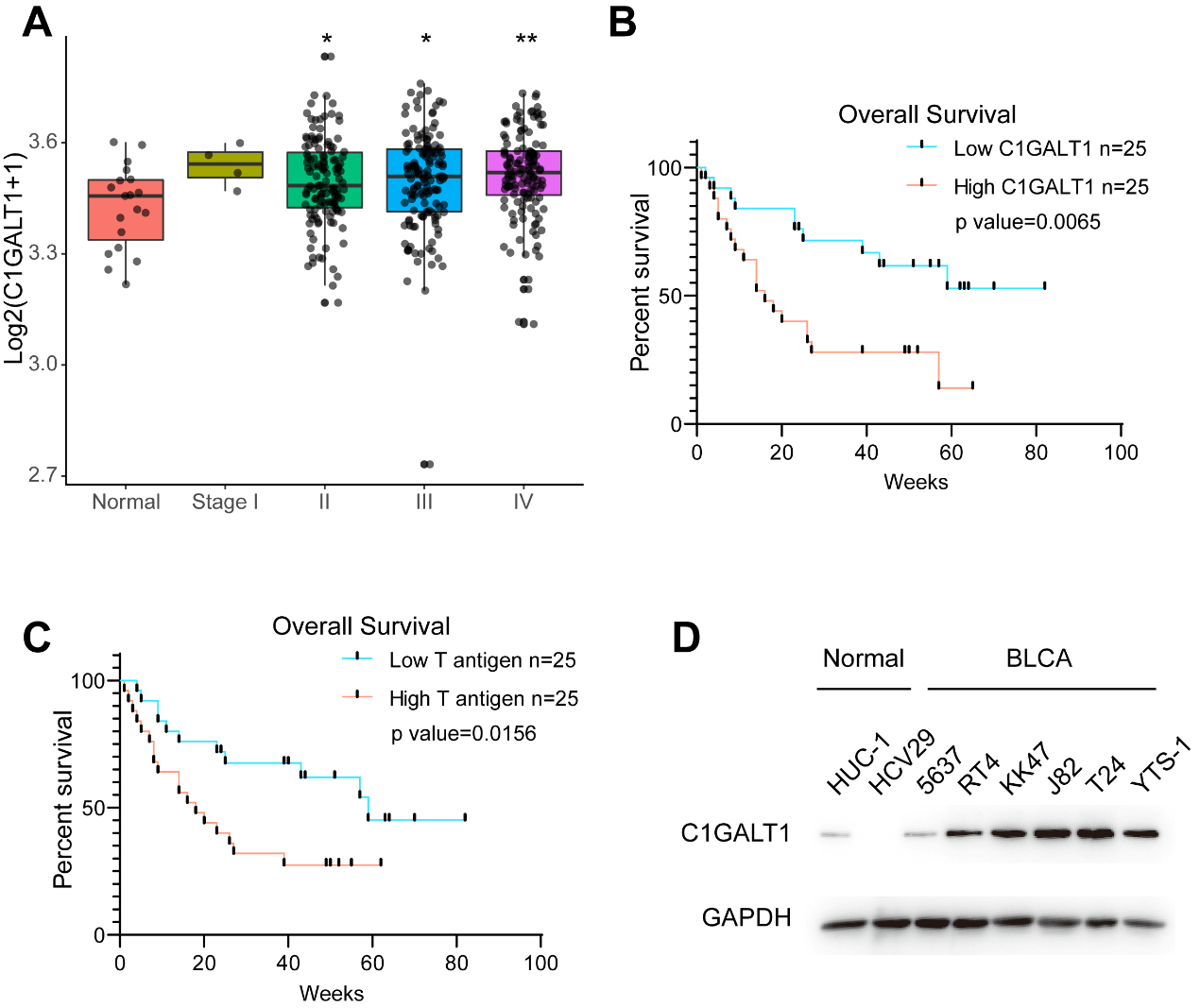


Fig. S2. Effects of C1GALT1-silencing on malignant behavior of YTS-1 and T24 cells

**(A)** T antigen levels in C1GALT1-silenced YTS-1 cells by flow cytometry. **(B)** FGFR3 expression in C1GALT1-silenced YTS-1 cells. **(C)** Doxorubicin resistance of control and C1GALT1-silenced YTS-1 cells. **(D)** C1GALT1 expression in C1GALT1 silenced T24 cells. **(E, F)** Proliferation (E) and migratory ability (F) of C1GALT1-silenced YTS-1 cells. **(G)** C1GALT1 expression in ITZ-treated YTS-1 cells. **(H-K)** Proliferation (H), colony formation (I), migratory ability (J), and doxorubicin resistance (K) of ITZ-treated YTS-1 vs. control cells.


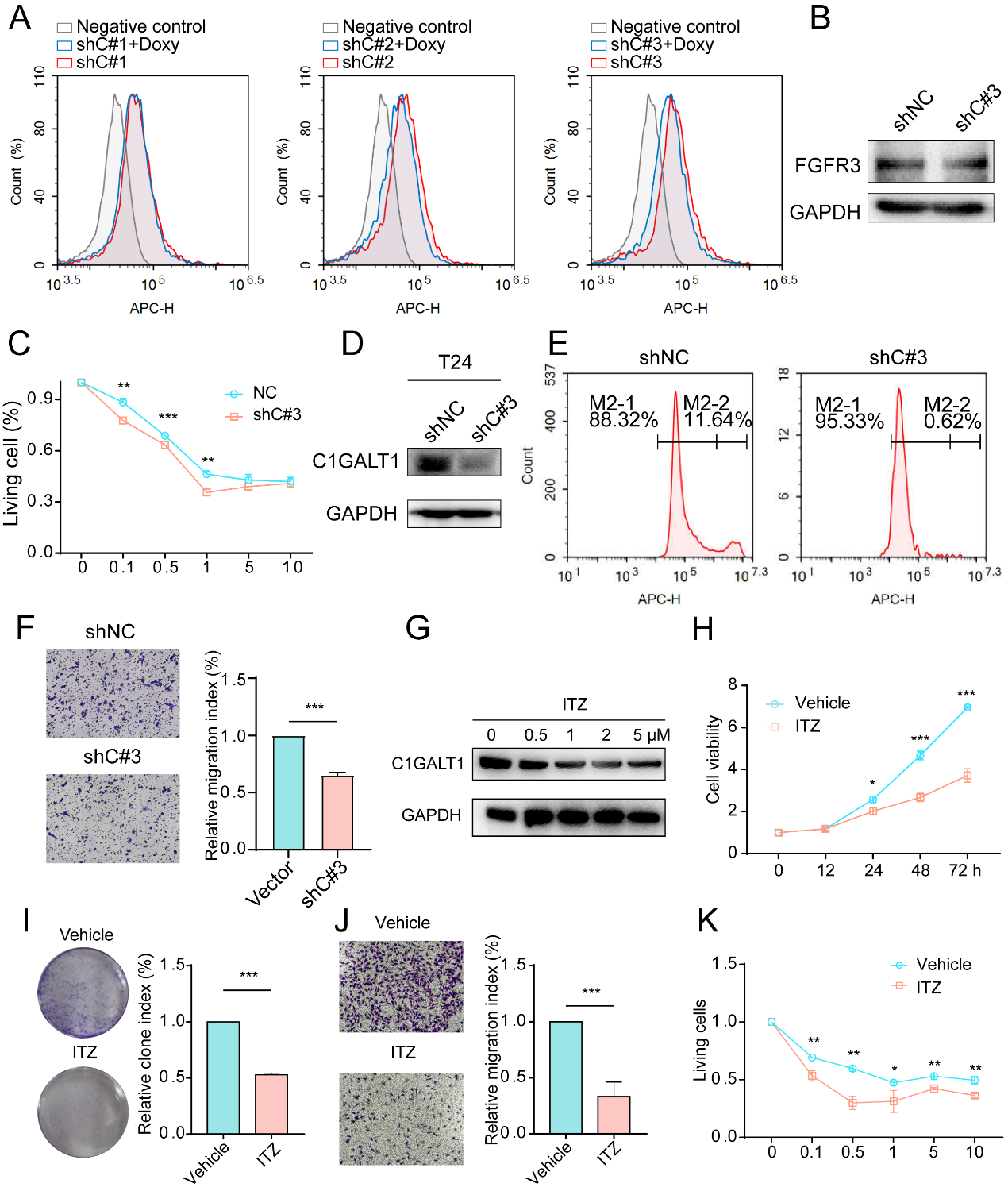


Fig. S3. Effects of C1GALT1-overexpressing on malignant behavior of HCV29 cells

**(A)** C1GALT1 expression in C1GALT1-overexpressing HCV29 cells. **(B-D)** Proliferation (B), colony formation (C) and migratory ability (D) of C1GALT1-overexpressing HCV29 cells.


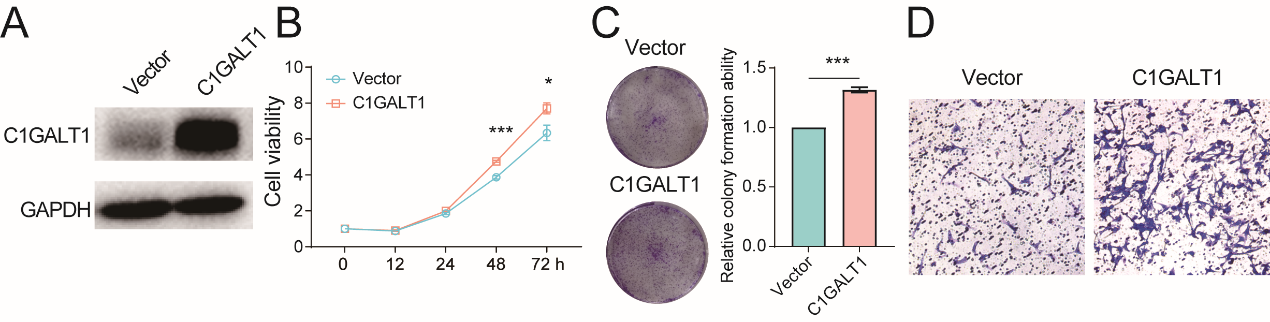


Fig. S4. Immunohistochemical analysis of tumors of mice model

**(A)** Immunohistochemical analysis of Ki67, TUNEL, C1GALT1, and T antigen in tumors of mouse injected with C1GALT1-silenced, ITZ-treated, and control YTS-1 cells. **(B)** Immunohistochemistry analysis of Ki67, TUNEL and C1GALT1 in PDXs with or without ITZ treatment.


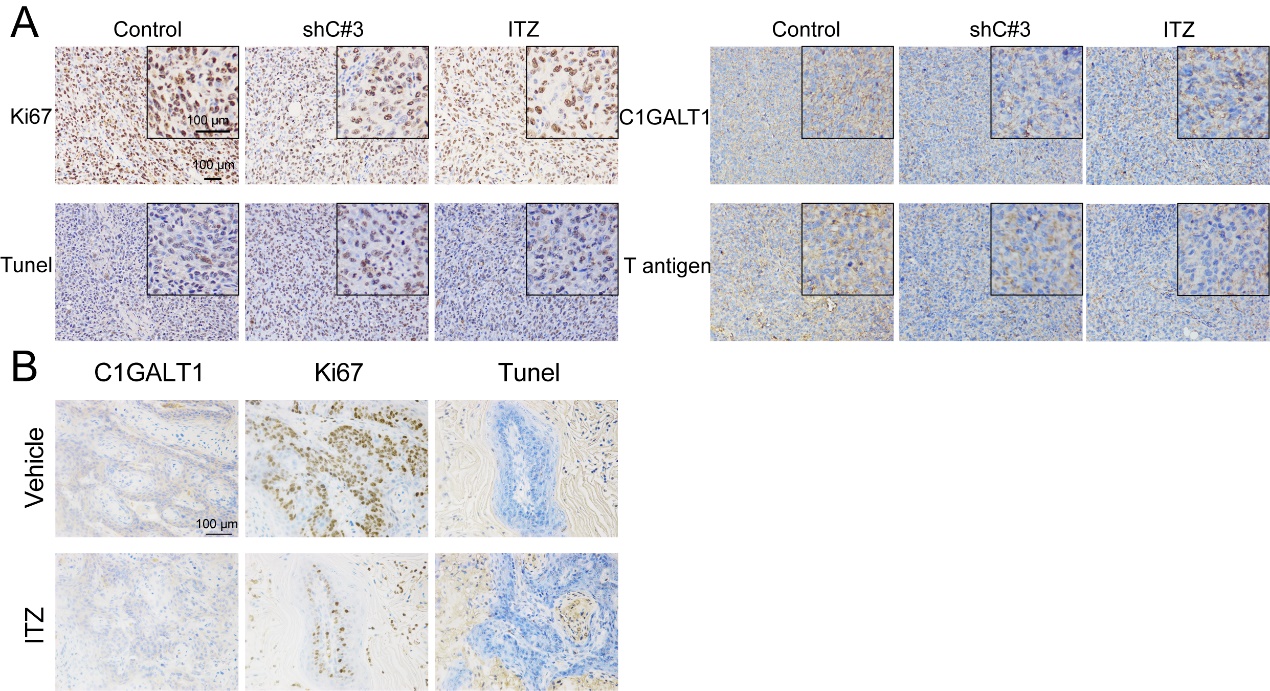


Fig. S5. Immunohistochemical analysis of C1GALT1, T antigen, Ki67, and TUNEL in tumors of trans-splenic metastasis model mice injected with control and C1GALT1-silenced YTS-1 cells


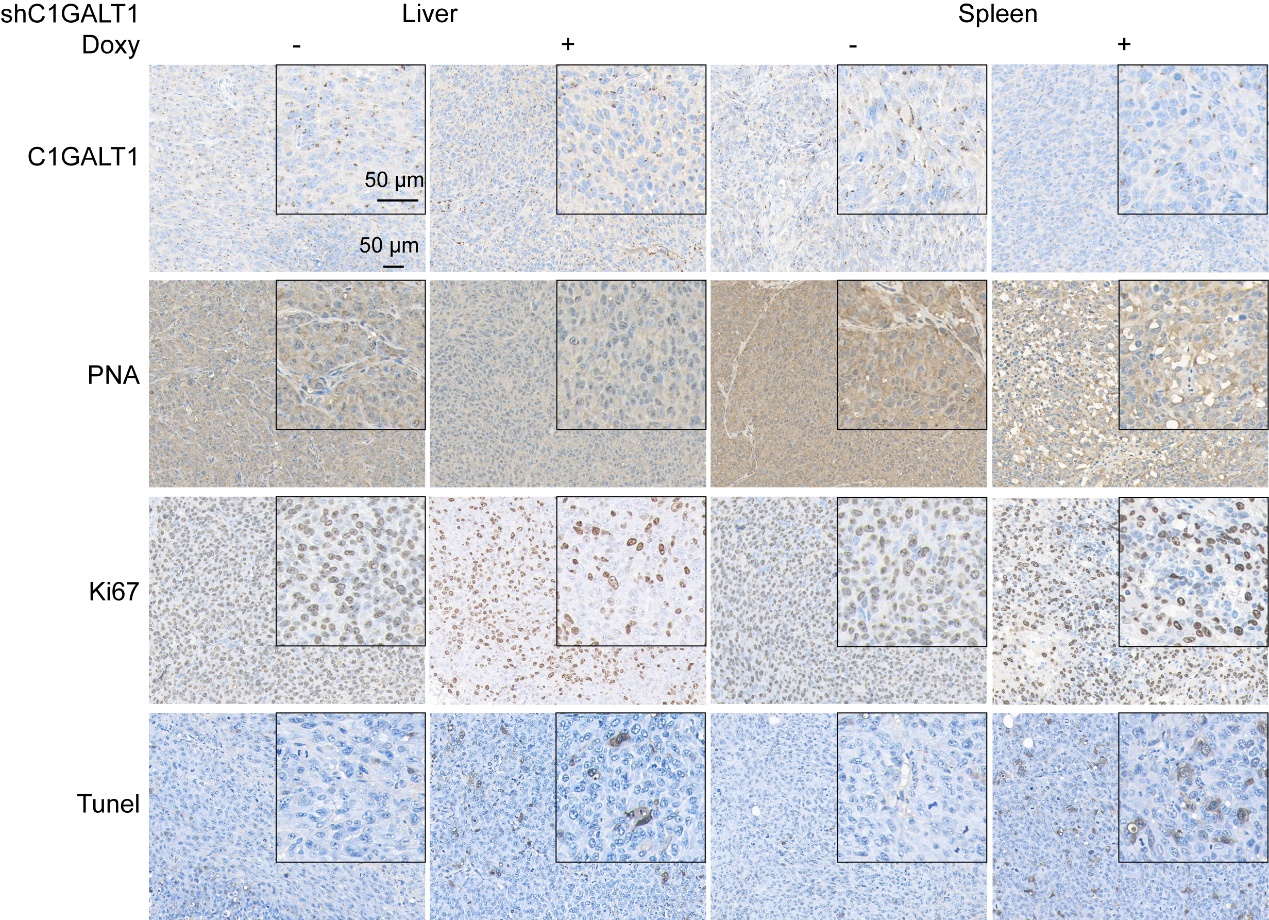


Fig. S6. H&E staining and immunohistochemistry analysis of LNs of popliteal lymphatic metastasis mouse model

**(A, B)** H&E staining (A) and immunohistochemistry analysis (B) of LNs of popliteal lymphatic metastasis model mouse injected with C1GALT1-silenced and control YTS-1 cells into footpads.


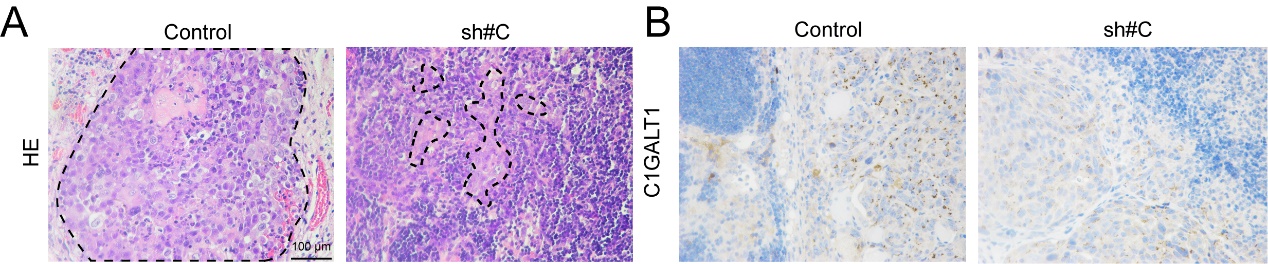


Fig. S7. Effects of miR-1-3p overexpressing on malignant behavior of BLCA YTS-1 cells

**(A)** C1GALT1 expression in miR-1-3p-overexpressing T24 cells. **(B, C)** Proliferation (B) and migratory ability (C) of miR-1-3p-overexpressing T24 cells. **(D)** Doxorubicin resistance of control and miR-1-3p-overexpressing YTS-1 cells. **(E)** C1GALT1 and T antigen levels of YTS-1 cells transfected with miR-1-3p mimic or inhibitors. **(F, G)** Proliferation (F) and migratory ability (G) of YTS-1 cells transfected with miR-1-3p mimic or inhibitor, in comparison with control cells. **(H)** Immunohistochemical analysis of Ki67, TUNEL, C1GALT1, and T antigen in tumors of mouse injected with miR-1-3p-overexpresssing and control YTS-1 cells.


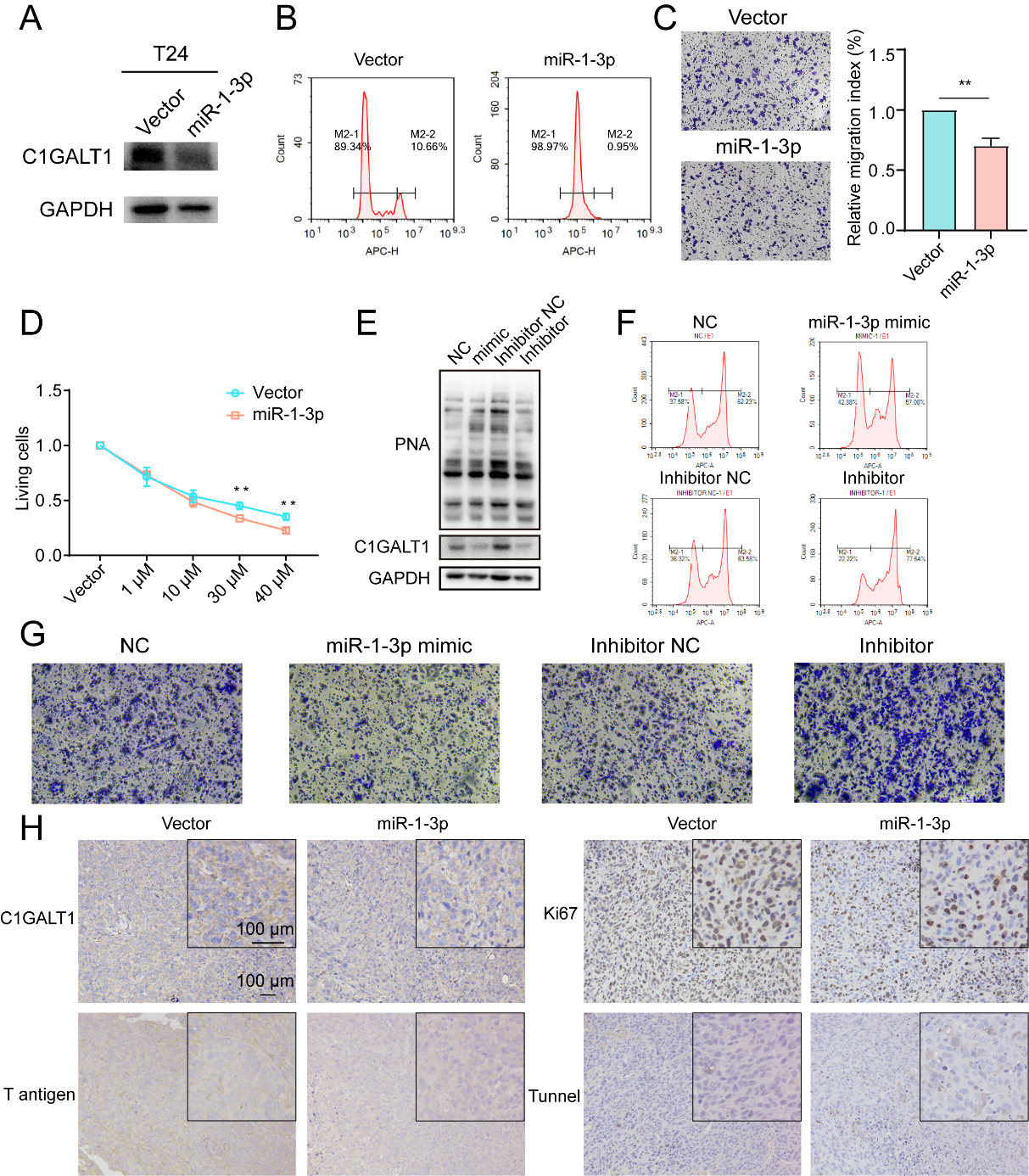


Fig. S8. Immunohistochemical analysis of C1GALT1, T antigen, Ki67, and TUNEL in tumors of trans-splenic metastasis model mice injected with control and miR-1-3p-overexpressing YTS-1 cells


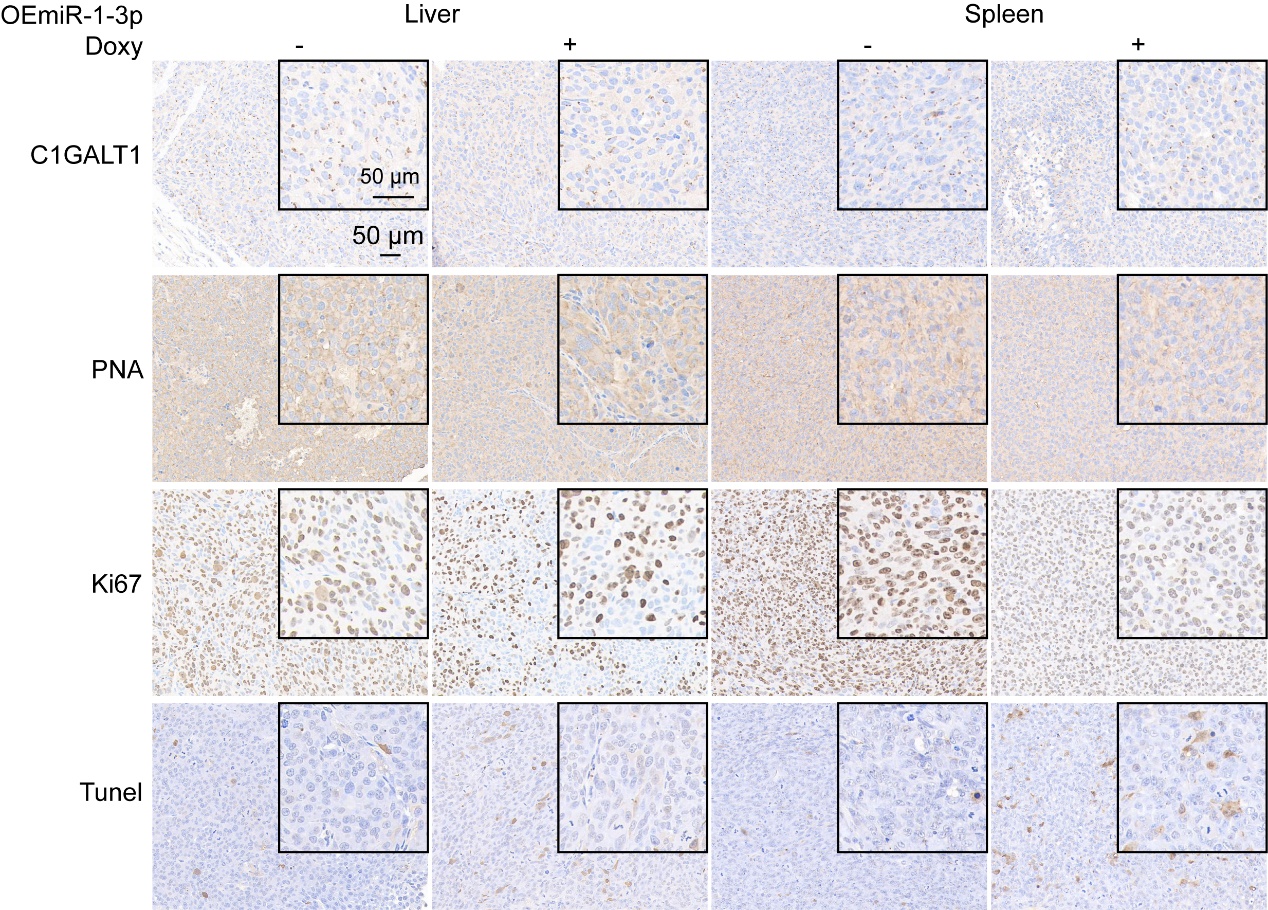


Fig. S9. Confirmation of circRNA backsplice junctions by Sanger sequencing

**(A)** Sequences of backsplice junctions of selected circRNAs. Arrows: backsplice sites. **(B)** Production of cHP1BP3 (schematic).


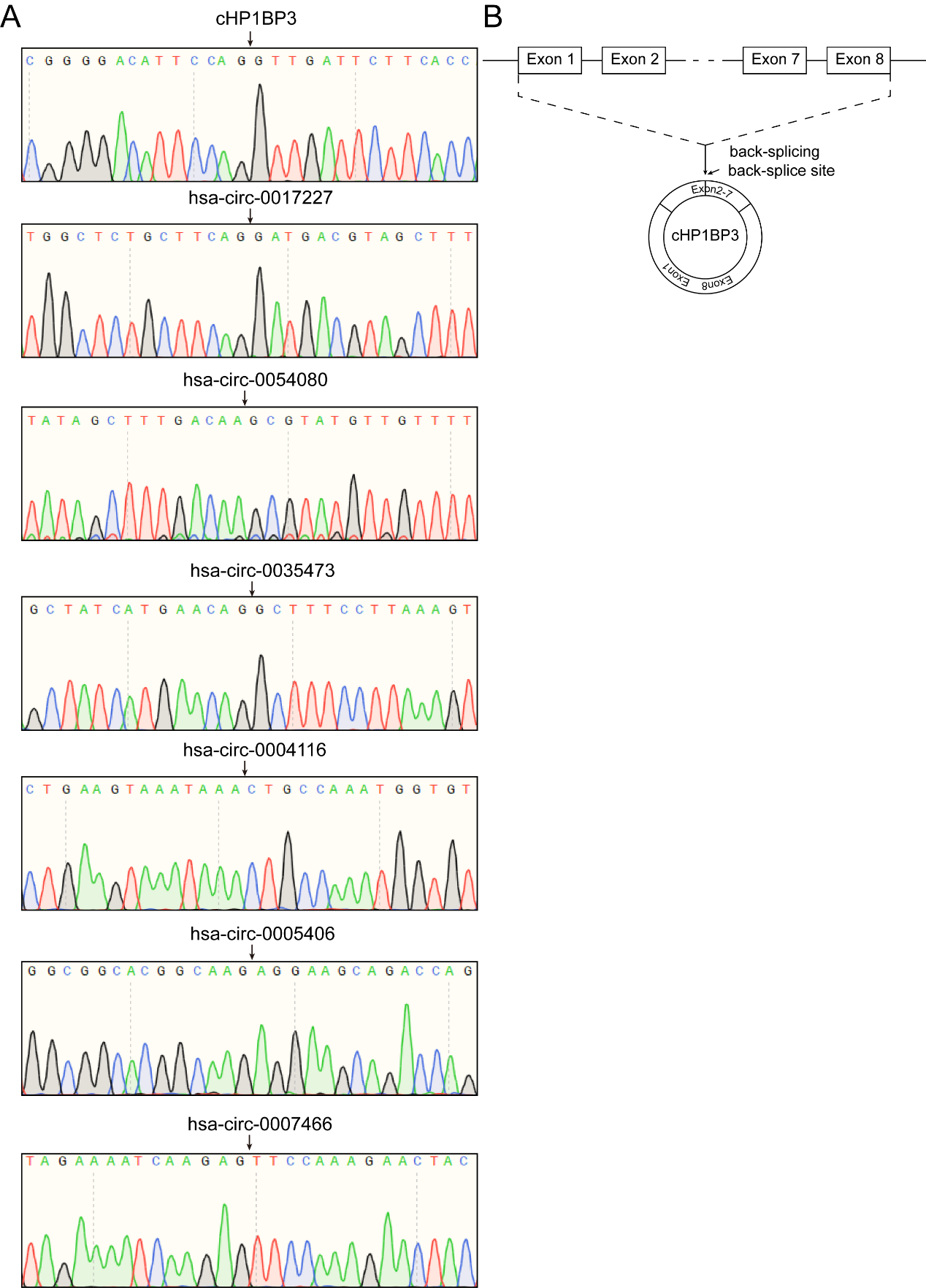


Fig. S10. Expression of circular cHP1BP3 and its linear counterpart in BLCA cells

**(A)** Sequence of mutated cHP1BP3 binding sites targeted by miR-1-3p. **(B)** RNA FISH for cHP1BP3 in BLCA and adjacent normal tissues. Nuclei were stained with DAPI, and miR-1-3p was FITC-labeled. **(C)** Expression of linear counterpart of cHP1BP3 in BLCA patients, in TCGA database.


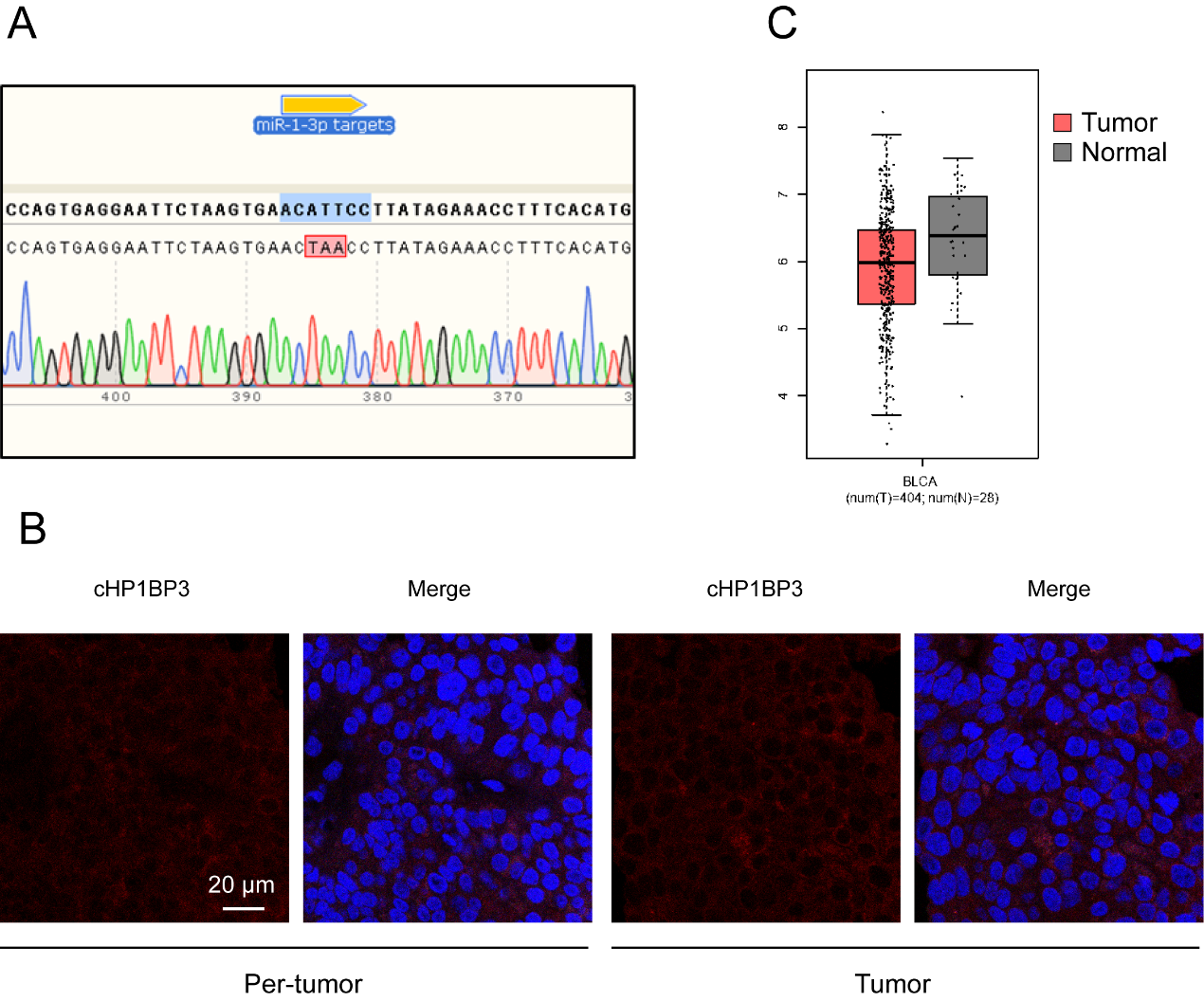


Fig. S11. Effects of cHP1BP3-silencing on malignant behavior of HCV29 and T24 cells

**(A-C)** Expression of cHP1BP3 (A), C1GALT1, and T antigen (B, C) in cHP1BP3-silenced YTS-1 and T24 cells. **(D)** Doxorubicin resistance of control and cHP1BP3-silenced YTS-1 cells. **(E, F)** Proliferation (E) and migratory ability (F) of cHP1BP3-silenced T24 cells. **(G)** Immunohistochemical analysis of Ki67, TUNEL, C1GALT1, and T antigen in tumors of mouse injected with control and cHP1BP3-silenced YTS-1 cells.


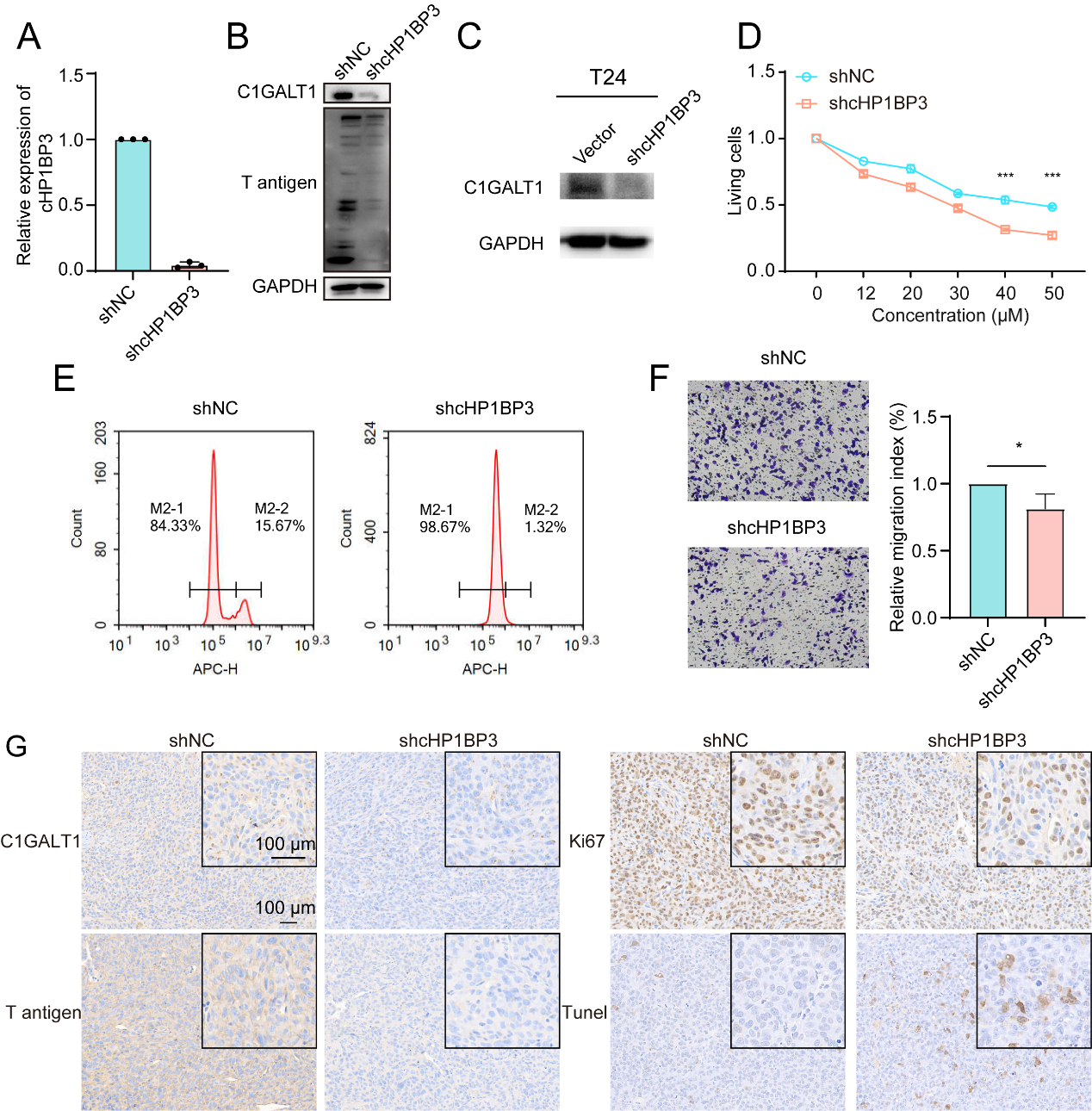


Fig. S12. Immunohistochemical analysis of C1GALT1, T antigen, Ki67, and TUNEL in tumors of trans-splenic metastasis model mice injected with control and cHP1BP3-silenced YTS-1 cells


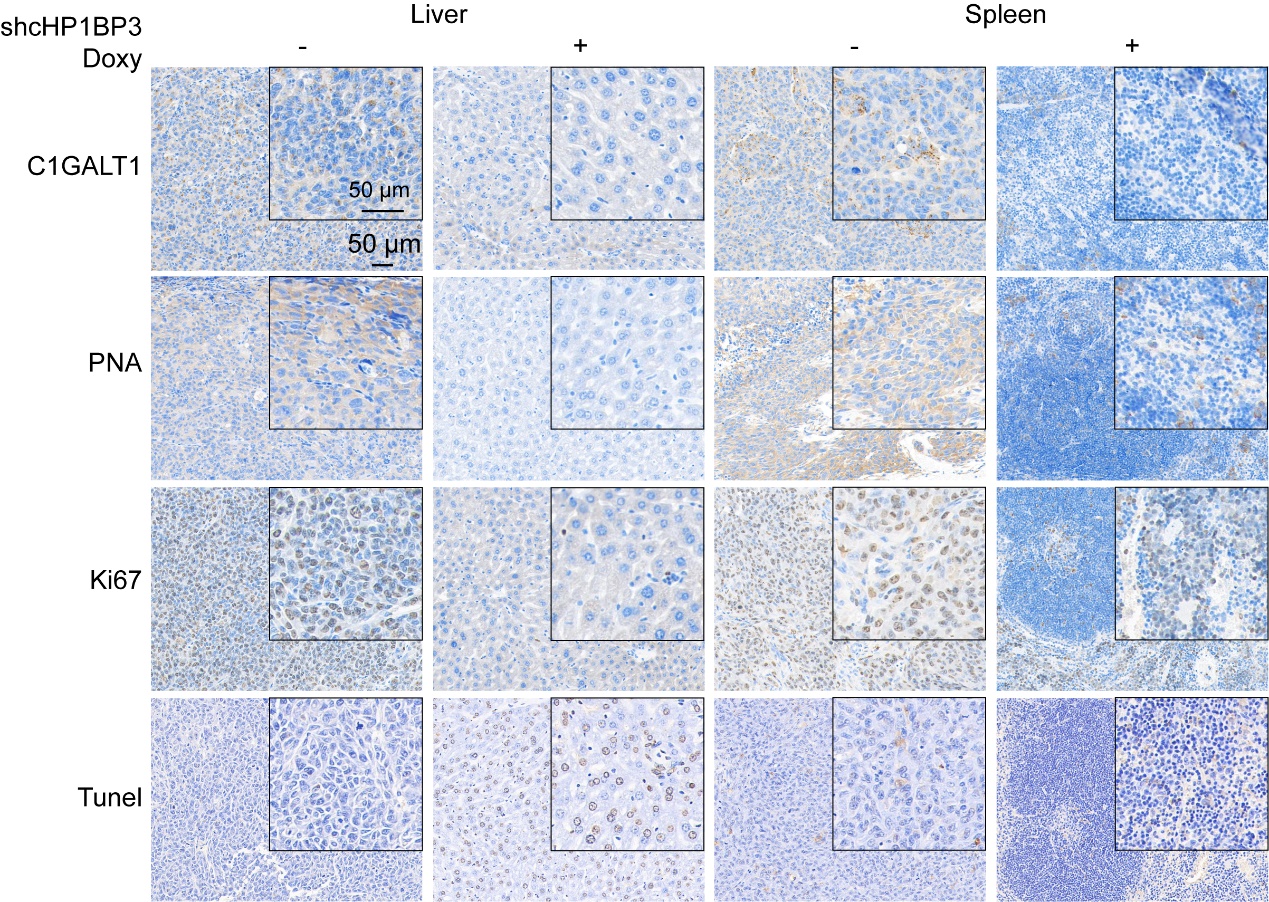

Supplement: Supplementary file 6 — Additional file 6: Fig. S1. Expression of C1GALT1 in BLCA cells and tissues. A C1GALT1 mRNA expression in various stages of BLCA patients, in TCGA database. B-C Overall survival of dichotomized C1GALT1 (B) and T antigen (C) expression in BLCA patients using TMA. D C1GALT1 expression and T antigen levels in various BLCA (5637, RT4, KK47, J82, T24, YTS-1) and normal uroepithelial (HCV29, HUC-1) cell lines. Fig. S2. Effects of C1GALT1 silencing on malignant behavior of YTS-1 and T24 cells. (A) T antigen levels in C1GALT1-silenced YTS-1 cells by flow cytometry. (B) FGFR3 expression in C1GALT1-silenced YTS-1 cells. (C) Doxorubicin resistance of control and C1GALT1-silenced YTS-1 cells. (D) C1GALT1 expression in C1GALT1 silenced T24 cells. (E, F) Proliferation (E) and migratory ability (F) of C1GALT1-silenced YTS-1 cells. (G) C1GALT1 expression in ITZ-treated YTS-1 cells. (H-K) Proliferation (H), colony formation (I), migratory ability (J), and doxorubicin resistance (K) of ITZ-treated YTS-1 vs. control cells. Fig. S3. Effects of C1GALT1-overexpressing on malignant behavior of HCV29 cells. (A) C1GALT1 expression in C1GALT1-overexpressing HCV29 cells. (B-D) Proliferation (B), colony formation (C) and migratory ability (D) of C1GALT1-overexpressing HCV29 cells. Fig. S4. Immunohistochemical analysis of tumors of mice model. (A) Immunohistochemical analysis of Ki67, TUNEL, C1GALT1, and T antigen in tumors of mouse injected with C1GALT1-silenced, ITZ-treated, and control YTS-1 cells. (B) Immunohistochemistry analysis of Ki67, TUNEL and C1GALT1 in PDXs with or without ITZ treatment. Fig. S5. Immunohistochemical analysis of C1GALT1, T antigen, Ki67, and TUNEL in tumors of trans-splenic metastasis model mice injected with control and C1GALT1-silenced YTS-1 cells. Fig. S6. H&E staining and immunohistochemistry analysis of LNs of popliteal lymphatic metastasis mouse model. (A, B) H&E staining (A) and immunohistochemistry analysis (B) of LNs of popliteal lymphatic metastasis model m [file 13046_2022_2438_MOESM6_ESM.docx]
